# Supplementary figures and images for: Site-directed M2 proton channel inhibitors enable synergistic combination therapy for rimantadine-resistant pandemic influenza
Source: PLoS Pathog. 2020 Aug 11;16(8):e1008716. doi: 10.1371/journal.ppat.1008716 (PMC7418971; doi:10.1371/journal.ppat.1008716)

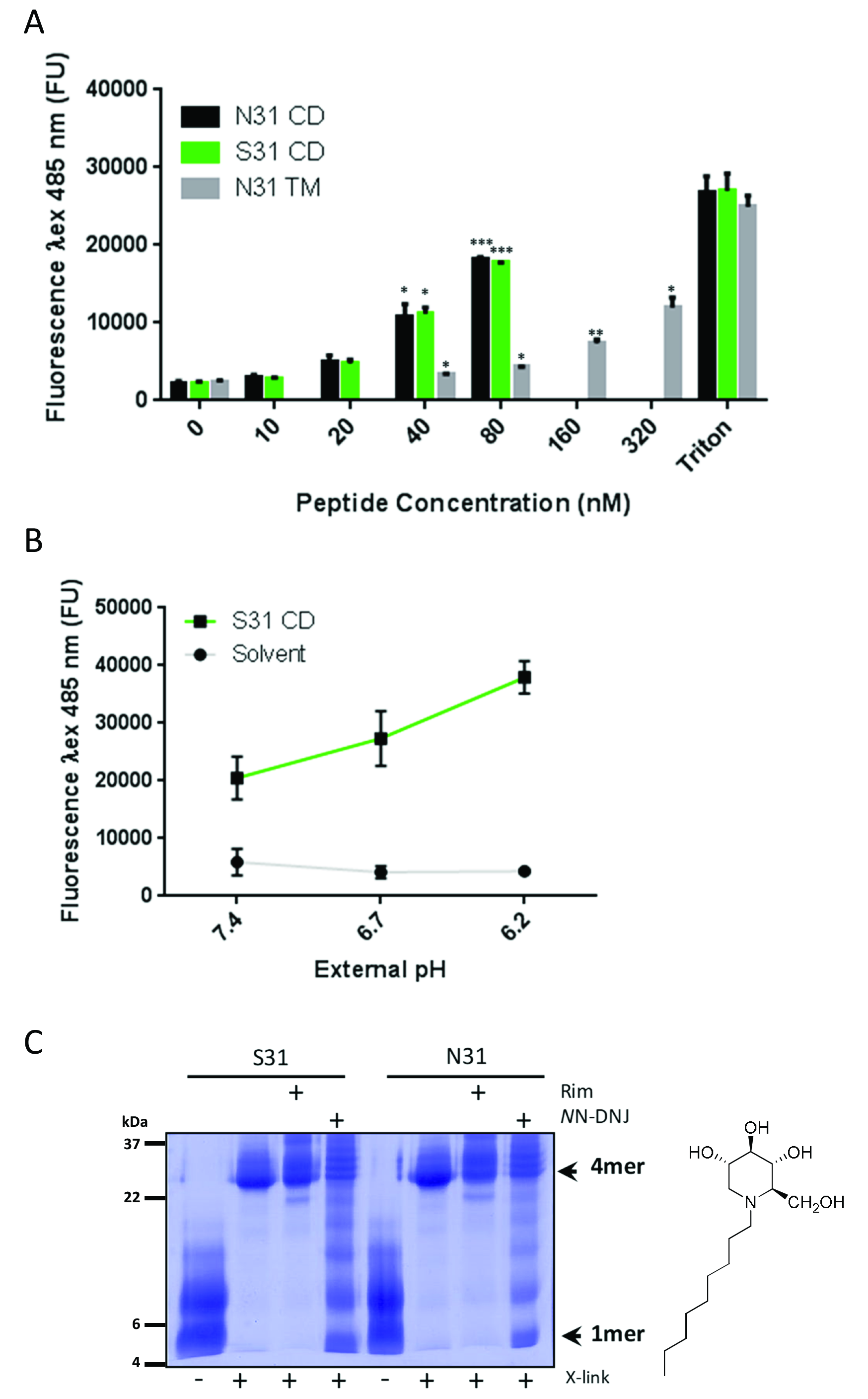

Supplement: S1 Fig — A. titration of biological activity for Eng195 M2 CD (N31/S31) and TM peptides. TM peptides form channels less efficiently compared with CD, thereby requiring 320 nM compared with 40 nM for equivalent biological activity at each experimental condition. B. Biological activity of M2 peptides in liposome release assay is responsive to acidic pH. End-point assays were conducted in alternate pH buffers prior to removal of liposomes by ultracentrifugation and re-buffering of reactions to restore CF fluorescence, as described previously. C. Disruption of M2 oligomerisation by alkylated imino-sugars. M2-N31 and M2-S31 CD peptides were incubated with liposomes in the presence of 40 μM rimantadine or NN-DNJ, prior to the addition of DSP (Lomant’s reagent). Resultant M2 complexes were visualised using SDS-Urea gel electrophoresis. (TIF) [file ppat.1008716.s001.tif]

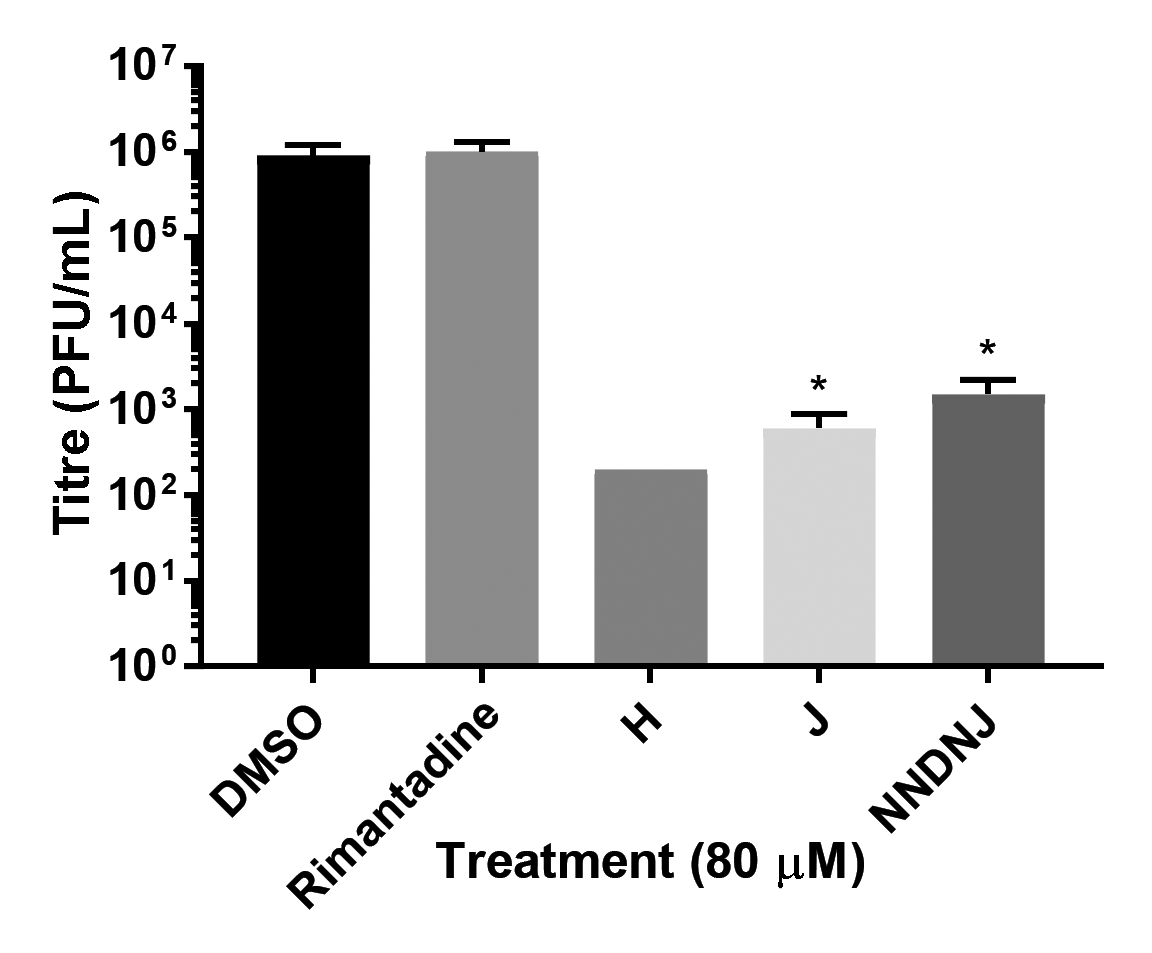

Supplement: S2 Fig — Compounds present at 80 μM both during infection (MOI of 0.01 pfu/cell) and through a 24 hr incubation in producer cells prior to titration of secreted infectivity. Rimantadine (80 μM) negative control was included. Data are representative of three technical repeats (* p≤0.05, paired student t-test). (TIF) [file ppat.1008716.s002.tif]

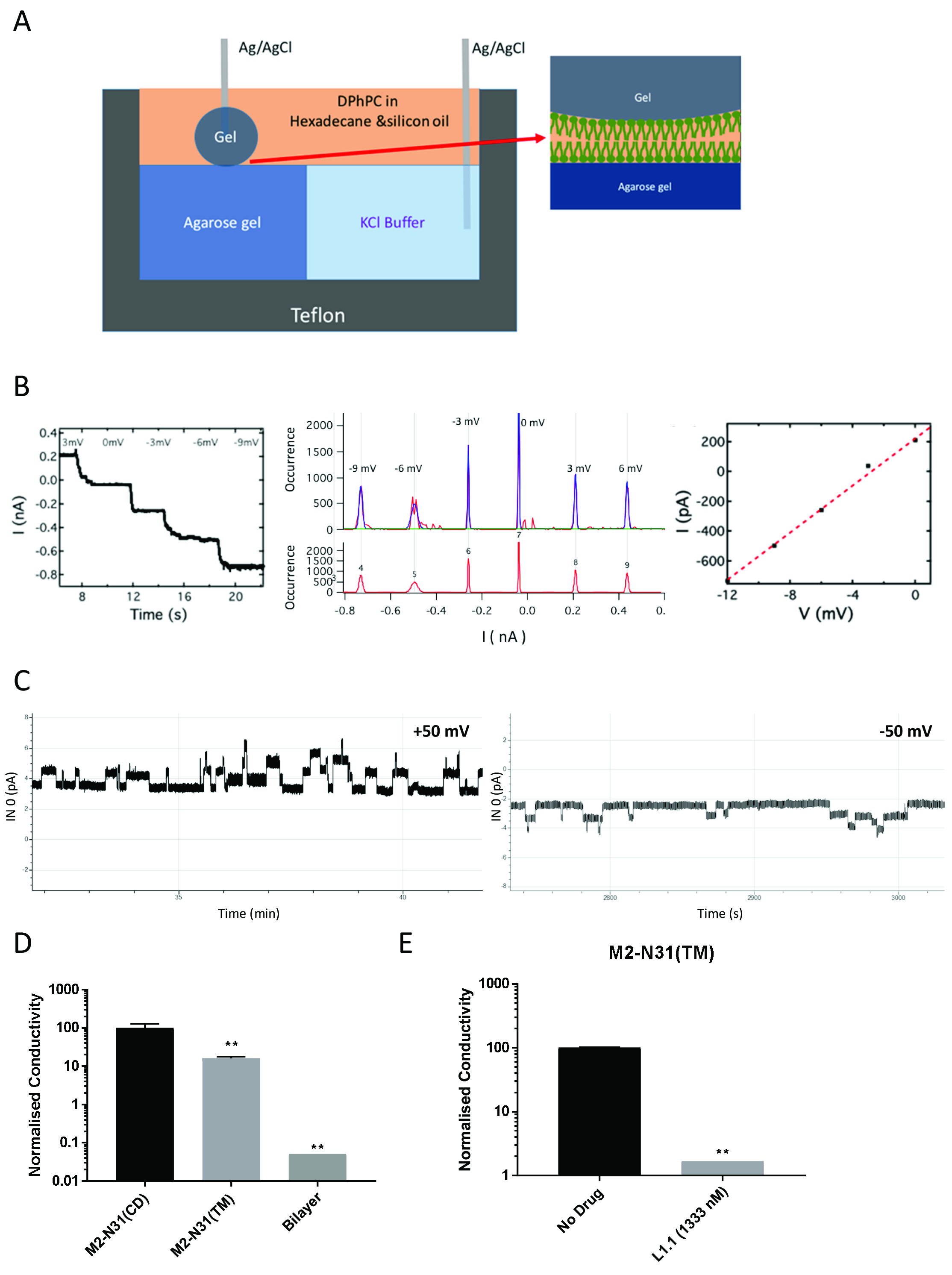

Supplement: S3 Fig — A. Organisation of the in-house Teflon holder rig used to create gel-gel interface bilayers as described in materials and methods. Inset shows orientation and formation of lipid bilayers. B. Example characterisation of bilayers harbouring M2 peptides showing the of current values at different voltages (left panel), plotted as a histogram fitted by multiple Gaussian peaks to determine the mean current values at different voltages (middle), allowing an I-V curve (right) to be plotted for use in calculating normalised bilayer conductance. C. Single channel recordings of M2-N31 CD peptides at voltages of +/- 50 mV. D. Normalised conductance of M2-N31 CD versus TM peptides (2000 nM) taken from three independent experiments. Error bars represent standard error of the mean with p values determined using the student T-test (* p≤0.05, ** p≤0.01). E. Inhibition of TM peptides (2000 nM) using L1.1 (1333 nM) taken from three independent experiments. Error bars represent standard error of the mean with p values determined using the student T-test (* p≤0.05, ** p≤0.01). (TIF) [file ppat.1008716.s003.tif]

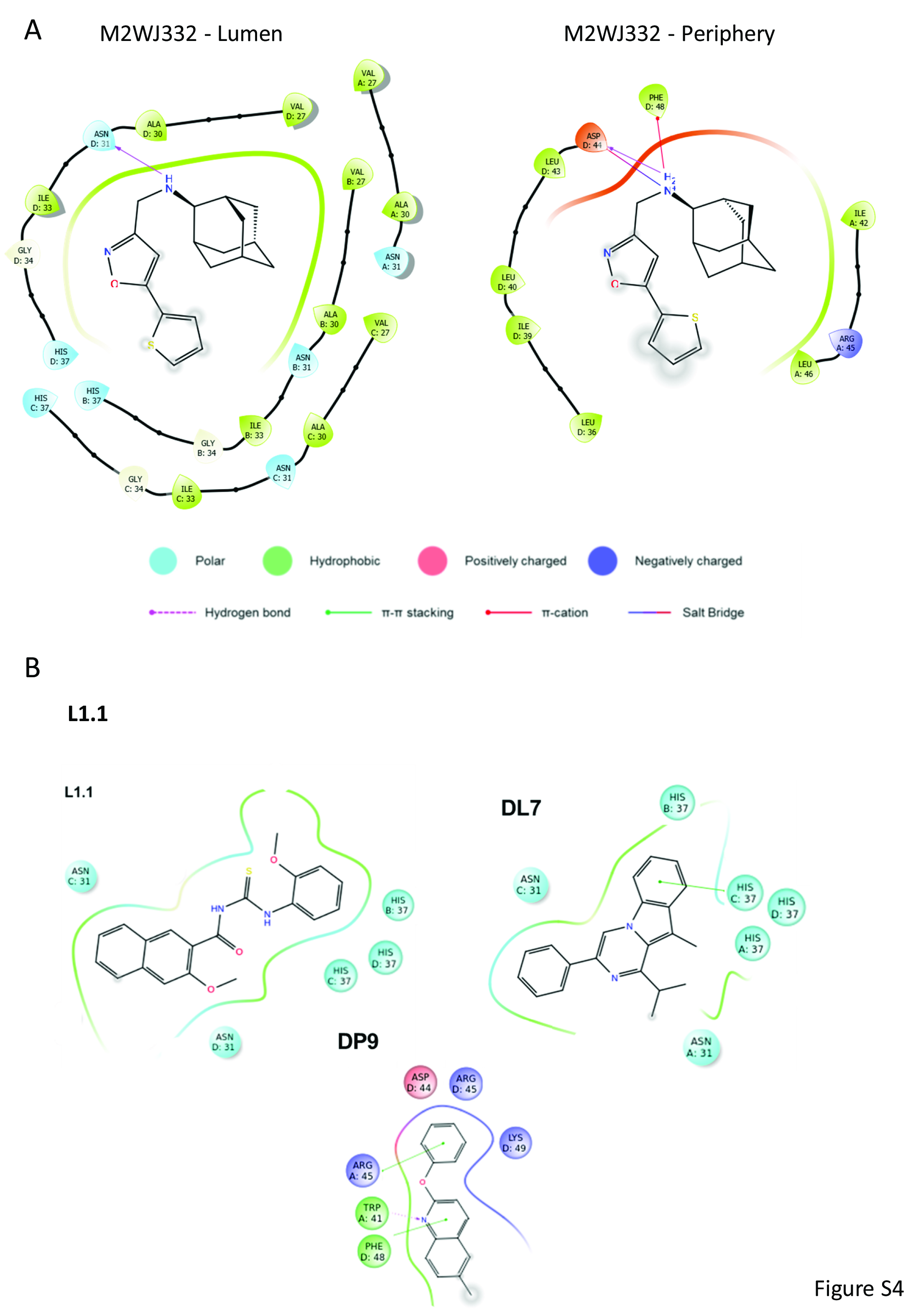

Supplement: S4 Fig — A. Predicted interactions between M2WJ332 and the Eng195 (E195) homology model when docked into the lumen (left) and the periphery (right). Docking studies conducted using Glide. B. As for A, but detailing L1.1 (Lumen), DL7 (lumen) and DP9 (periphery). (TIF) [file ppat.1008716.s004.tif]

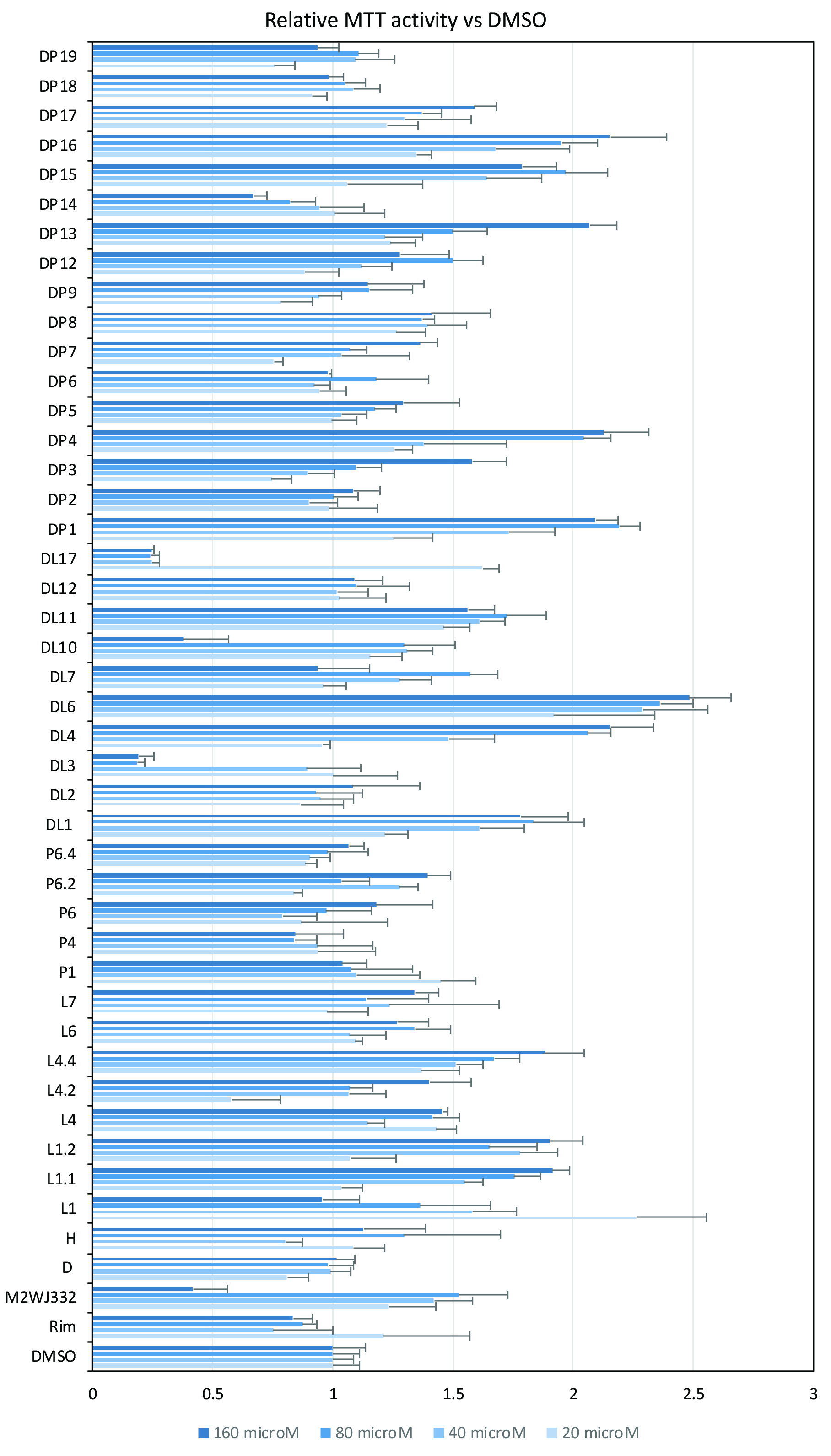

Supplement: S5 Fig — Compounds showing significant activity versus M2-N31 in vitro were assessed for potential cytotoxic effects. MDCK cells were incubated with increasing compound concentrations (20–160 μM) for 24 h prior to assessing metabolic activity via MTT assay. Results are representative of at least two biological repeats, each containing four technical repeats. Error bars are standard deviations within one biological repeat. Compounds showing any sign of impaired metabolic effects at 40 μM or greater were discounted from virological screens. (TIF) [file ppat.1008716.s005.tif]

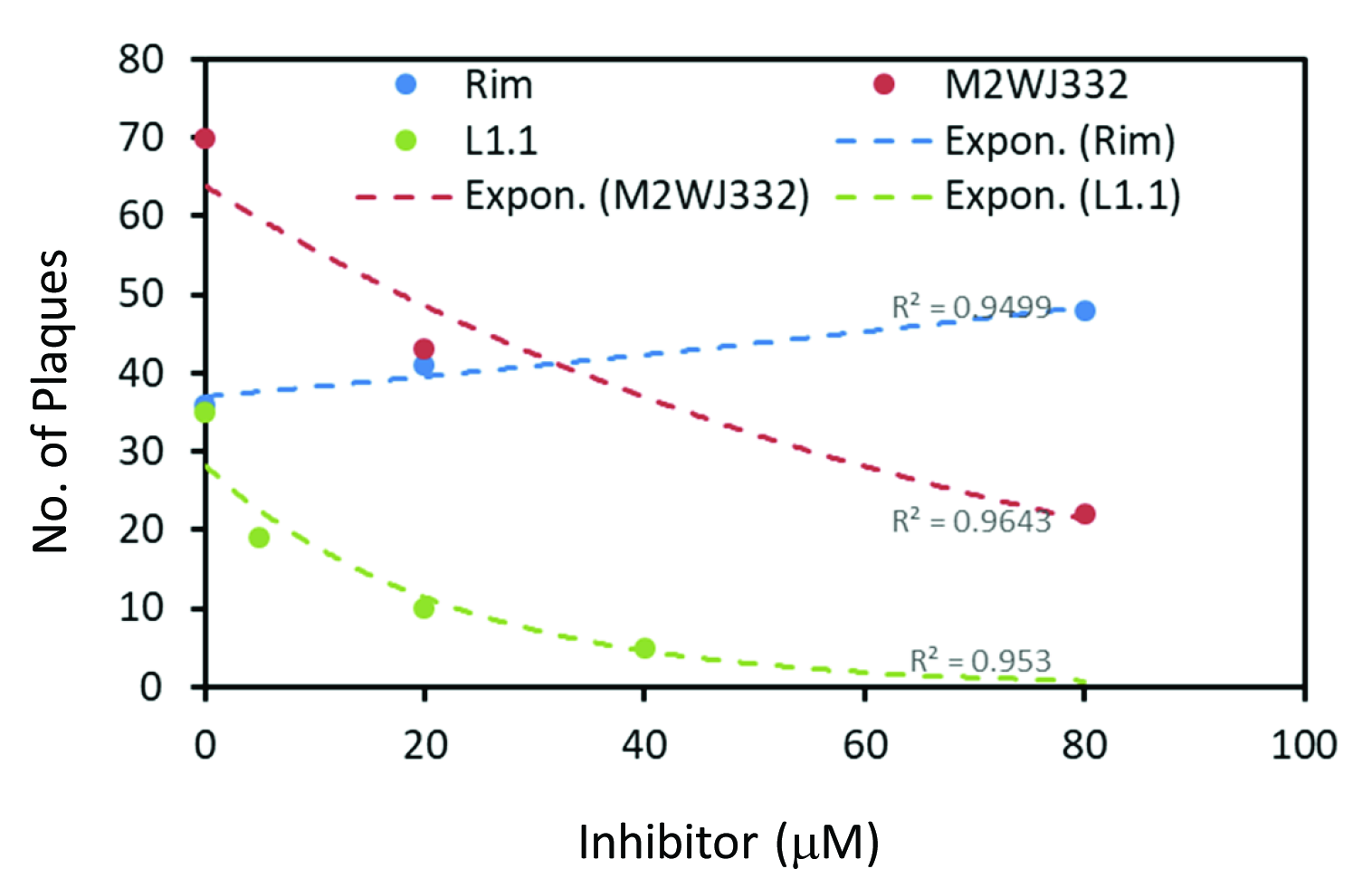

Supplement: S6 Fig — Normalised supernatants were diluted and plated onto naïve cells (triplicates in a 12-well plate) in the presence of increasing inhibitor concentrations for 1 h, after which media was replaced by media/soft agar containing the same inhibitor concentrations. Resultant plaques (mean from triplicate wells) were counted, plotted as a function of inhibitor concentration, and fitted to exponential curves in Excel with R2 values as shown. (TIF) [file ppat.1008716.s006.tif]

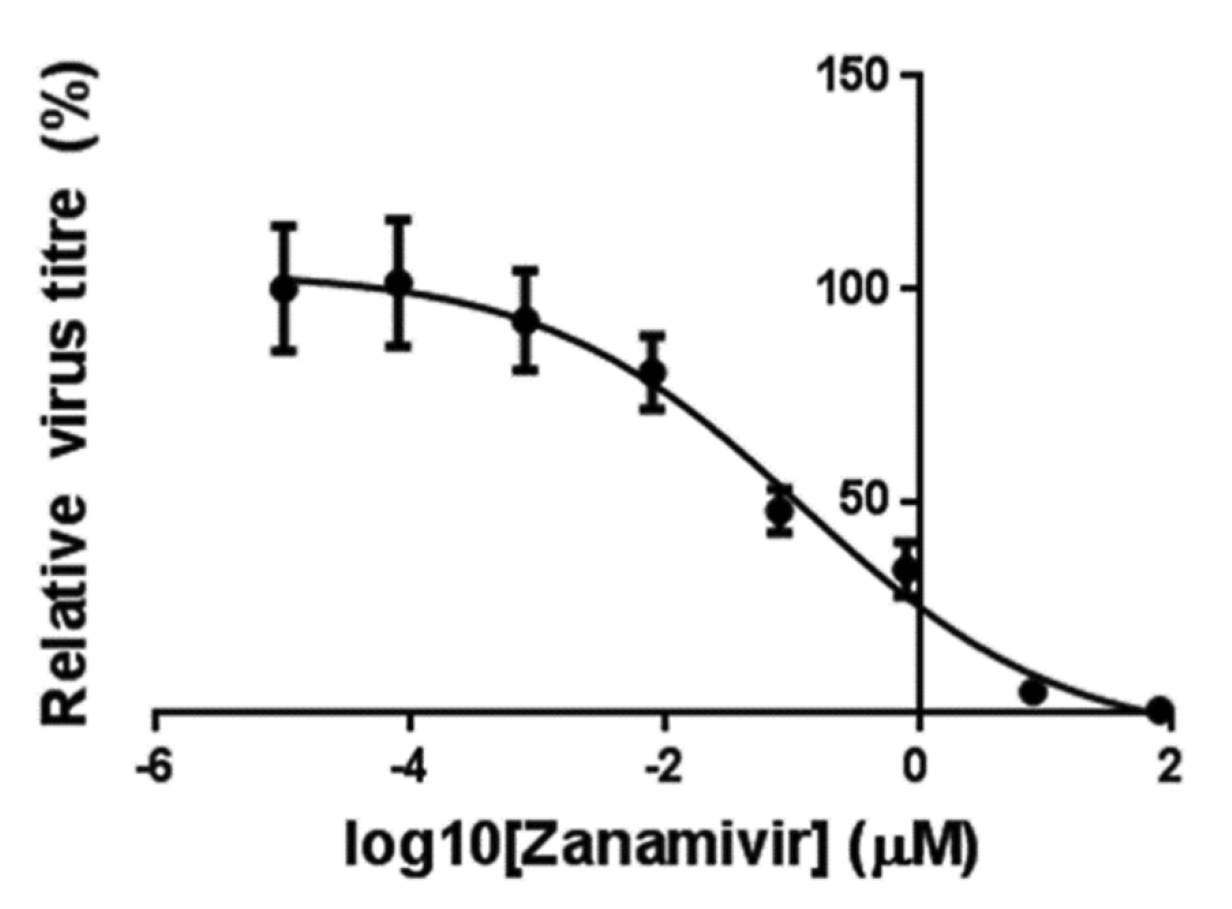

Supplement: S7 Fig — (TIF) [file ppat.1008716.s007.tif]

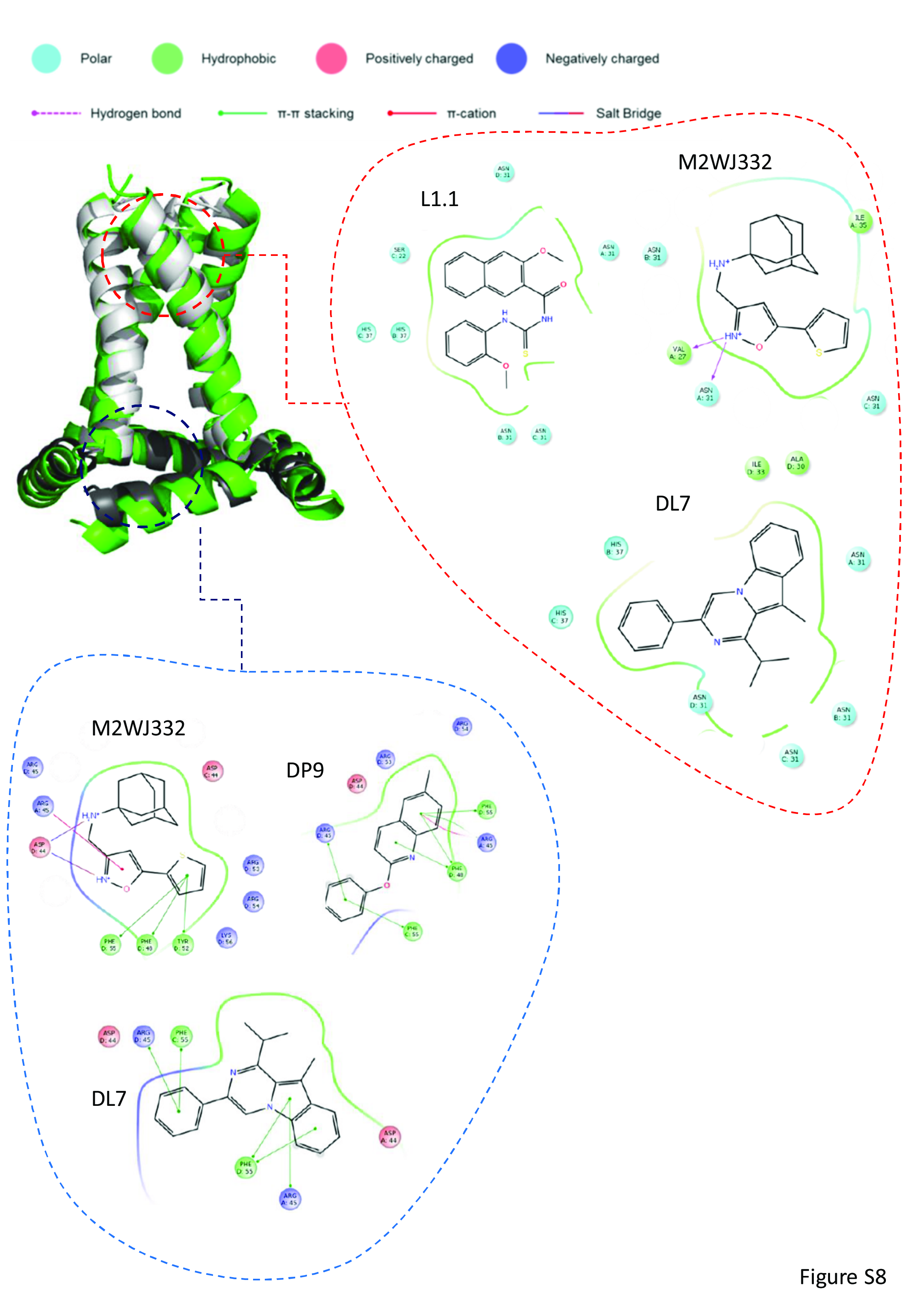

Supplement: S8 Fig — (TIF) [file ppat.1008716.s008.tif]
