# Supplementary material for: Site-directed M2 proton channel inhibitors enable synergistic combination therapy for rimantadine-resistant pandemic influenza
Source: PLoS Pathog. 2020 Aug 11;16(8):e1008716. doi: 10.1371/journal.ppat.1008716 (PMC7418971; doi:10.1371/journal.ppat.1008716)
Supplement: S1 Data — (PDF) [file ppat.1008716.s009.pdf]

PEPTIDES INTERNATIONAL

11621 Electron Drive
Louisville, Kentucky 40299 USA
Phone: 502-266-8787
Fax: 502-267-1FAX (1329)
peptides@pepnet.com
PEPNET.COM

ANALYTICAL DATA SHEET

Product Name: **H-RCSDSSDPLVIAASIIGILHLILWITDRLFFKCIYRRFKY GLK-NH_2**
H-Arg-Cys-Ser-Asp-Ser-Ser-Asp-Pro-Leu-Val-Ile-Ala-Ala-Ser-Ile-Ile-Gly-Ile-Leu-His-Leu-Ile-Leu-Trp-Ile-Thr-Asp-Arg-Leu-Phe-Phe-Lys-Cys-Ile-Tyr-Arg-Arg-Phe-Lys-Tyr-Gly-Leu-Lys-NH_2

Catalog No.	*PCS-30083-PI*	*Lot No.*	*919371*
Formula	$C_{236}H_{376}N_{62}O_{56}S_2$	*Molecular Weight*	*5042.14*

Appearance *White powder*

ES-MS *MW Calculated 5038.79* *MW Found 5038.13*

Amino Acid Analysis

Asp	*2.32*	*(3)*
Ser	*3.09*	*(4)*
Gly	*1.92*	*(2)*
His	*0.87*	*(1)*
Arg	*4.37*	*(4)*
Thr	*0.95*	*(1)*
Ala	*2.00*	*(2)*
Pro	*1.01*	*(1)*
Tyr	*1.90*	*(2)*
Val	*0.93*	*(1)*
Lys	*2.77*	*(3)*
Ile	*6.76*	*(7)*
Leu	*6.26*	*(6)*
Phe	*3.25*	*(3)*

Form *Trifluoroacetate Salt*

HPLC profile included (purity 93.6%)

Prepared by
Original Date: December 7, 2009
ldc
Revised Date: December 22, 2009
ldc

Approved by

Sample Information

Acquired by : Admin
Sample Name : PCS-30083-PI
Sample ID : 919371
Description : 32mg in10uLAcOH+60uL MeCN/TFA/H2O degassed

Vail# :
Injection Volume : 5 uL
Data Filename : 919371R.lcd
Method Filename : gradient 20_70.lcm
Batch Filename :
Report Filename : reportformat20_70.lcr
Date Acquired : 12/7/2009 10:29:34 AM
Data Processed : 12/7/2009 11:35:37 AM

Gradient: 20-70% in 50 min.
Column: Vydac C18, 218TP54, (4.6mm i.d., 250 mm L)
Buffer: A: 0.05% TFA in H2O B: 0.05% TFA in MeCN
Flow: 1mL/min

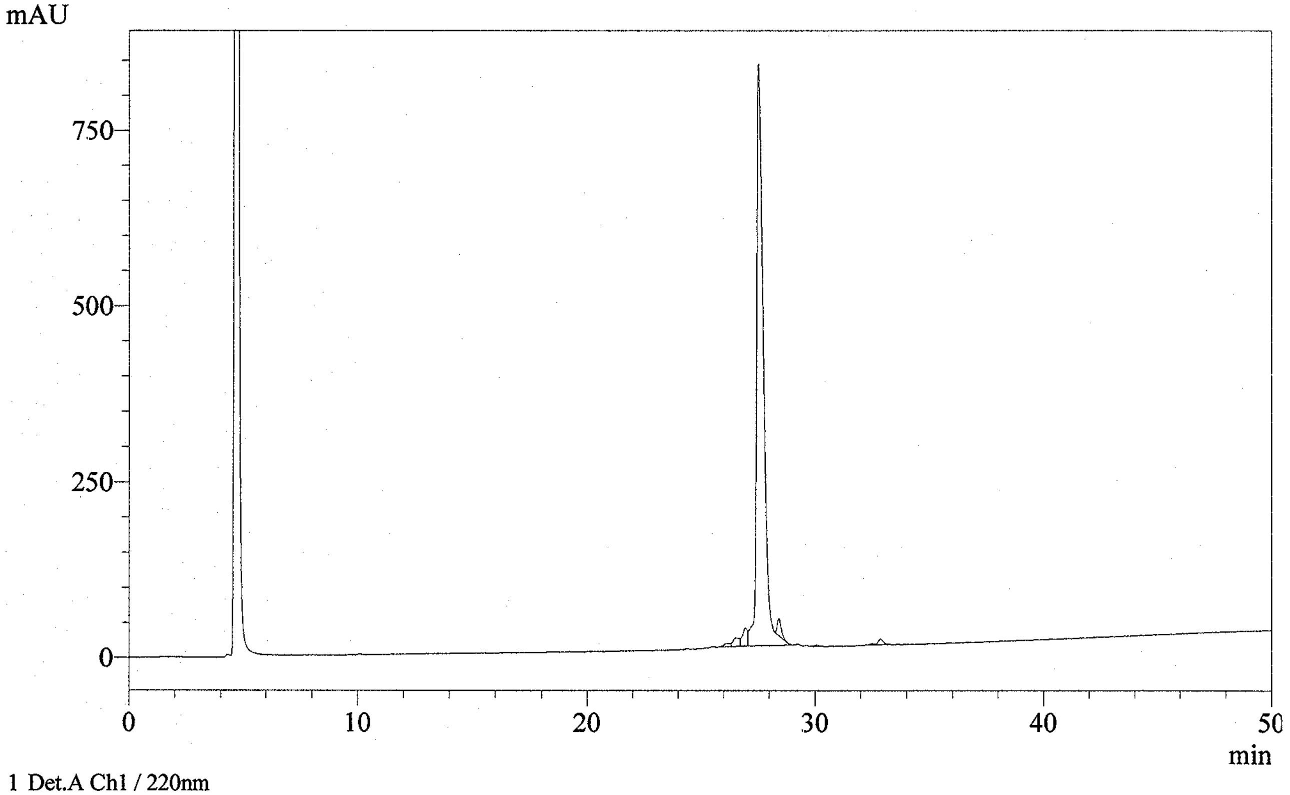

1 Det.A Ch1 / 220nm

PeakTable

Detector A Ch1 220nm

Ret. Time	Area	Height	Area %
26.108	90296	4716	0.485
26.517	251569	12012	1.350
26.953	397518	26277	2.133
27.530	17433096	827853	93.557
28.414	300037	24235	1.610
30.082	21396	1973	0.115
32.475	22670	1562	0.122
32.863	117145	7889	0.629
			100.000

Mariner Spec #1 ASC=>DECONV [BP = 5041.2, 4286]

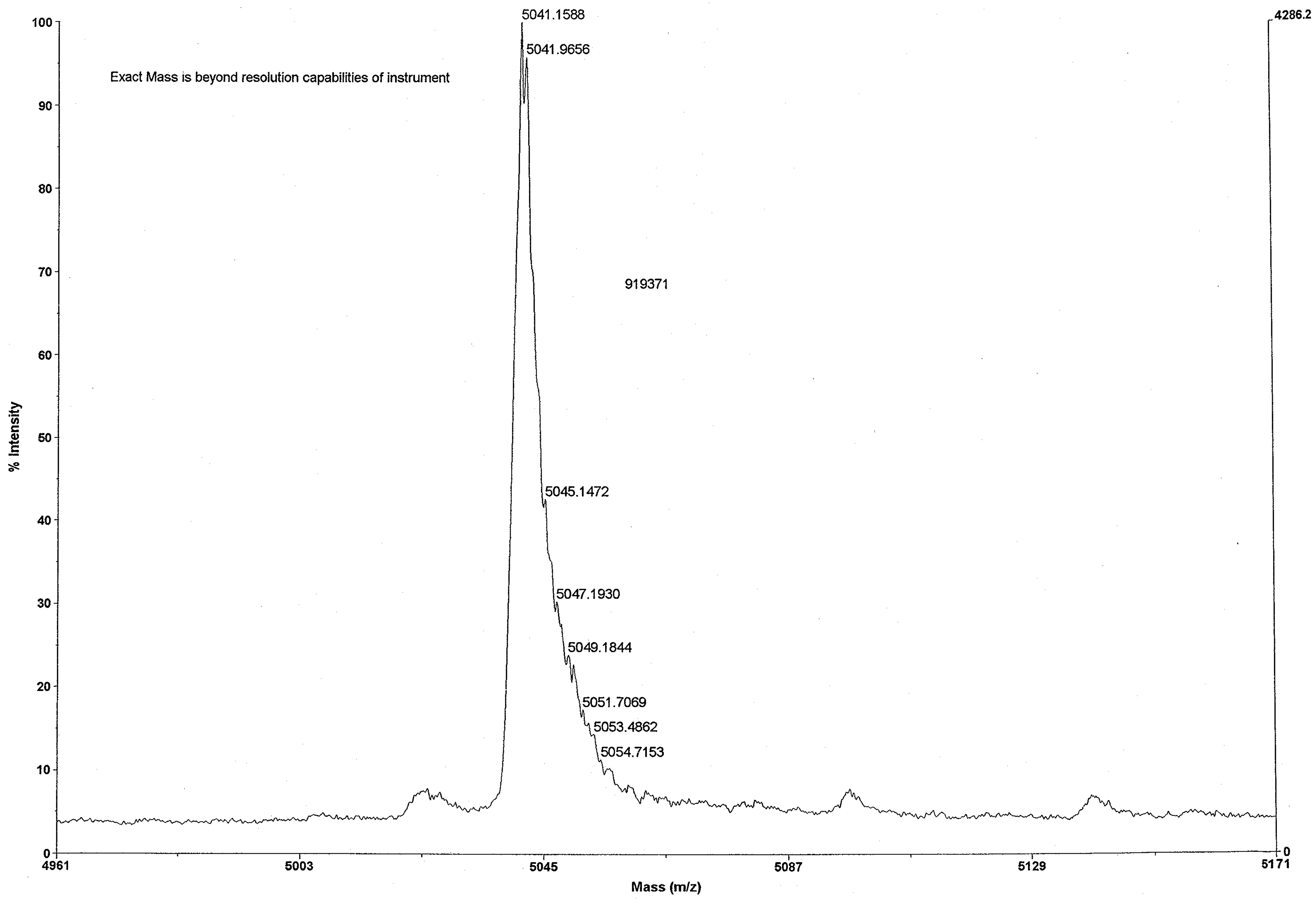

Mariner Mass Spectrum
D:\...\fv-19-37-1001.dat
Acquired: Dec 04 11:51:00 2009

Mariner Spec #1 ASC[BP = 721.2, 1347]

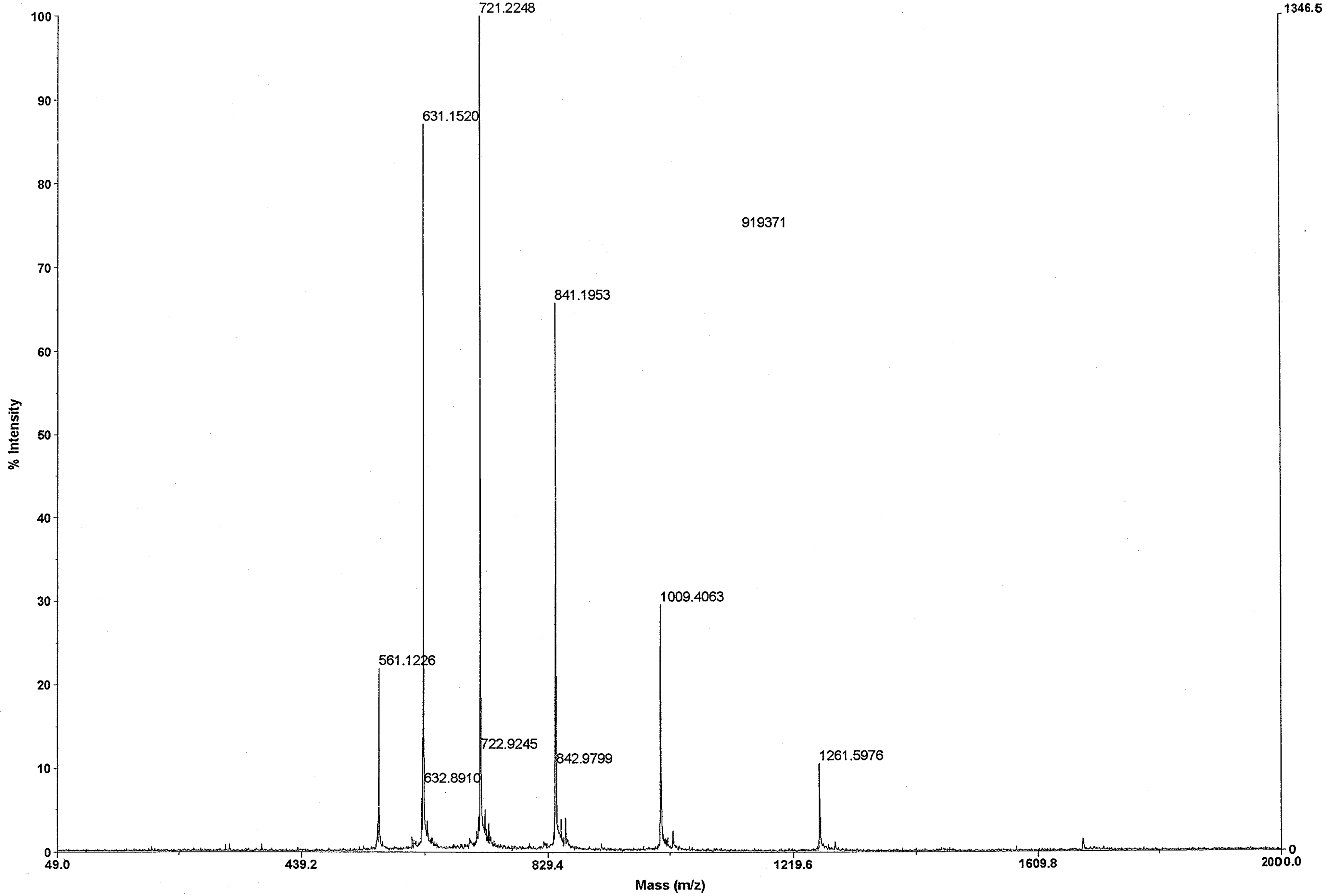

Mariner Mass Spectrum
D:\..\fv-19-37-1001.dat
Acquired: Dec 04 11:51:00 2009

PEPTIDES INTERNATIONAL

11621 Electron Drive
Louisville, Kentucky 40299 USA
Phone: 502-266-8787
Fax: 502-267-1FAX (1329)
peptides@pepnet.com
PEPNET.COM

ANALYTICAL DATA SHEET

Product Name: **H-RCSDSSDPLVIAANIIGILHLILWITDRLFFK CIYRRFKYGLK-NH_2**
H-Arg-Cys-Ser-Asp-Ser-Ser-Asp-Pro-Leu-Val-Ile-Ala-Ala-Asn-Ile-Ile-Gly-Ile-Leu-His-Leu-Ile-Leu-Trp-Ile-Thr-Asp-Arg-Leu-Phe-Phe-Lys-Cys-Ile-Tyr-Arg-Arg-Phe-Lys-Tyr-Gly-Leu-Lys-NH_2

Catalog No.	*PCS-30391-PI*	*Lot No.*	*000836C*
Formula	$C_{237}H_{377}N_{63}O_{56}S_2$	*Molecular Weight*	*5069.16*

Appearance *White powder*

ES-MS *MW Calculated* *5065.80* *MW Found* *5066.65*

Form *Trifluoroacetate Salt*

HPLC profile included (purity 96.9%)

Prepared by
Original Date: January 12, 2011
CM

Approved by

FORM PD6503
Rev01

Sample Information

Acquired by : Admin
Sample Name : PCS-30391-PI
Sample ID : 000836C
Description : 0.81mg in 43uL AcOH+200uL MeCN/TFA/H2O

Vail# : 0
Injection Volume : 3 uL
Data Filename : SYS1-000836CR.lcd
Method Filename : gradient40_75%.1cm
Date Acquired : 3/5/2012 2:04:00 PM
Data Processed : 3/5/2012 2:54:36 PM

Gradient: 40-75% in 35 min.
Column: Jupiter 5u, C18, 300A, 547029-24, (4.6mm x 250 mm)
Buffer: A: 0.05% TFA in H2O B: 0.05% TFA in MeCN
Flow: 1mL/min

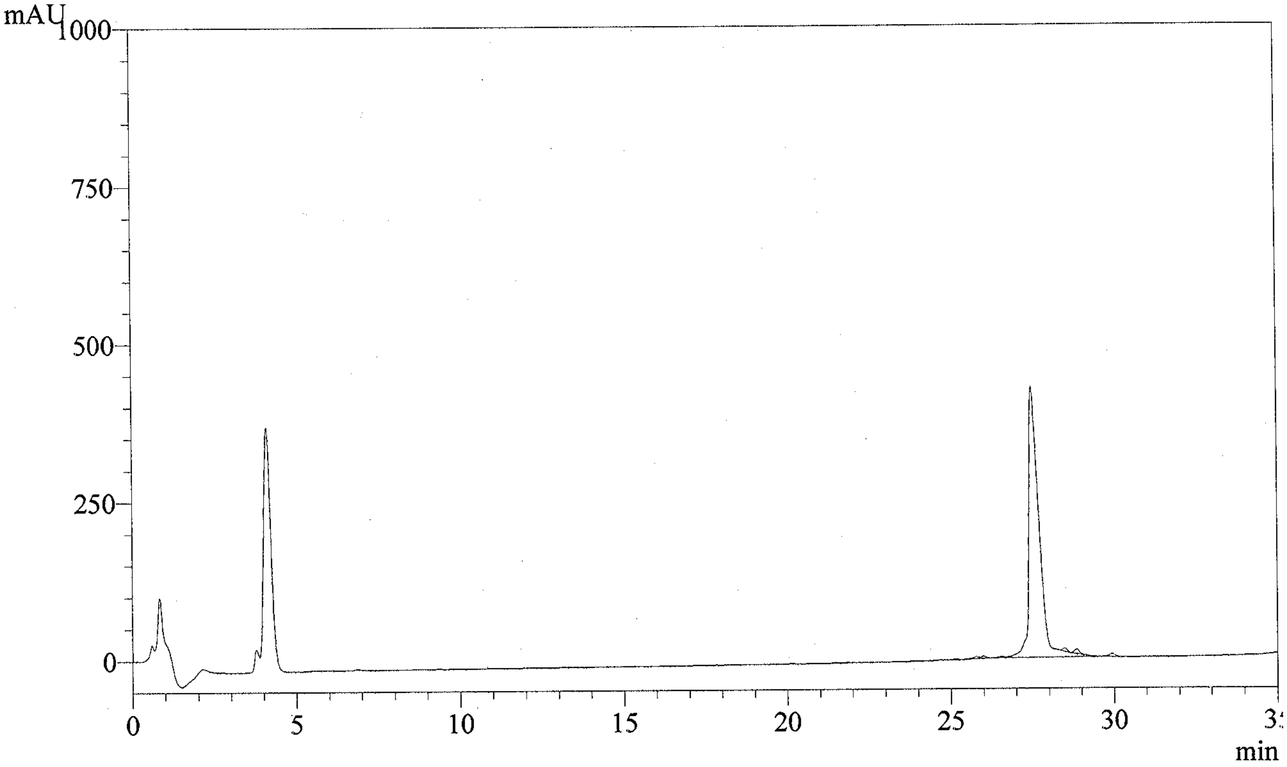

1 Det.A Ch1 / 220nm

PeakTable

Detector A Ch1 220nm

Ret. Time	Area	Height	Area %
21.475	5067	520	0.052
21.835	6890	770	0.071
25.127	16181	1433	0.167
25.770	44106	3306	0.454
25.992	35997	3263	0.371
26.523	24861	1961	0.256
27.488	9401934	429158	96.884
28.476	44116	4754	0.455
28.841	68696	6751	0.708
29.937	56489	4366	0.582
			100.000

Result Mass Spec=>SC[BP = 5068.7, 50]

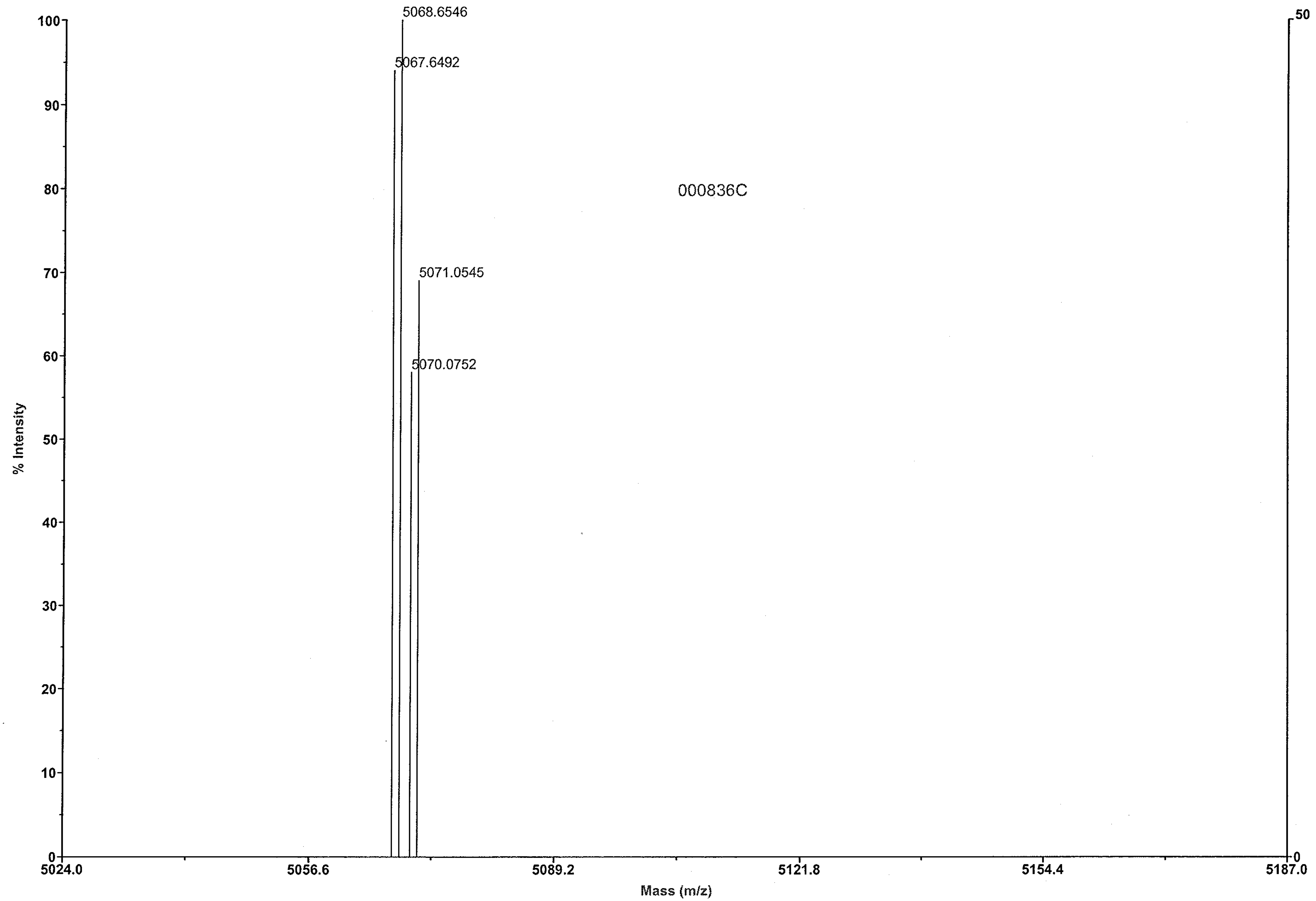

Mariner Spec #9928 [TIC = 42092, BP = 845.65, 72] from Mariner Instrument Control Panel using :C:\Mariner\Data\Factory\081511.bic*
D:\...\000836C.rst
Acquired: 14:43, March 05, 2012

Result Mass Spec[BP = 845.7, 53]

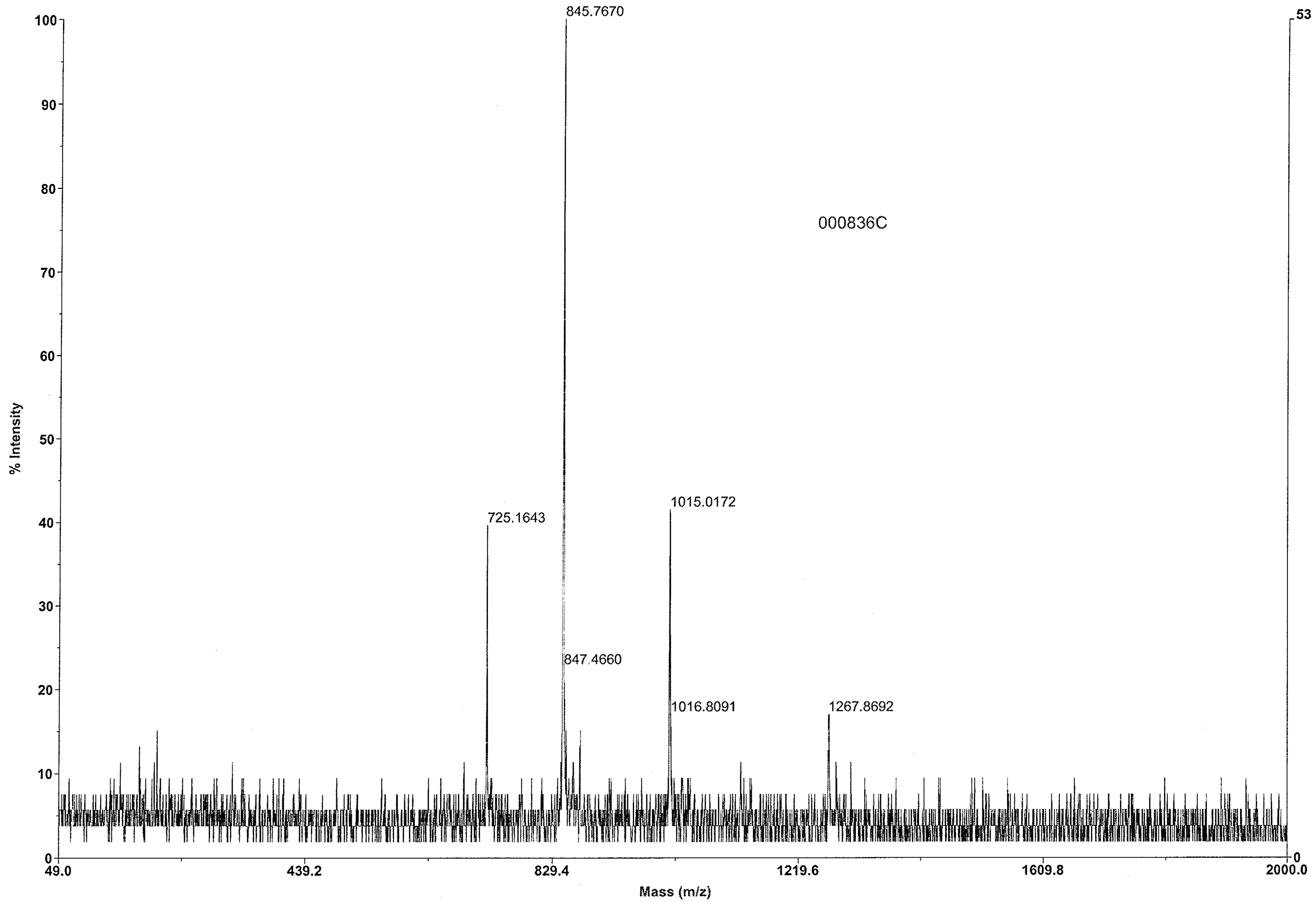

Mariner Spec #9928 [TIC = 42092, BP = 845.65, 72] from Mariner Instrument Control Panel using :C:\Mariner\Data\Factory\081511.bic*
D:\...\000836C.rst
Acquired: 14:43, March 05, 2012

PEPTIDES INTERNATIONAL

11621 Electron Drive
Louisville, Kentucky 40299 USA
Phone: 502-266-8787
Fax: 502-267-1FAX (1329)
peptides@pepnet.com
PEPNET.COM

ANALYTICAL DATA SHEET

Product Name: H-RCSDSSDPLVIAANIIGILHLILWITDRL-NH_2

H-Arg-Cys-Ser-Asp-Ser-Ser-Asp-Pro-Leu-Val-Ile-Ala-Ala-Asn-Ile-Ile-Gly-Ile-Leu-His-Leu-Ile-Leu-Trp-Ile-Thr-Asp-Arg-Leu-NH_2

Catalog No. *PCS-31319-PI* *Lot No.* *001926C*

Formula $C_{145}H_{242}N_{40}O_{40}S$

Appearance *White powder*

Molecular Weight Expected *3217.85* *Molecular Weight Found* *3219.03*

Form *Trifluoroacetate salt*

HPLC profile included (purity 95.3%)

Prepared by:
Manufacture Date: November 26, 2013
CM

Approved by

FORM PD6503
Rev 01

Sample Information

Acquired by : Admin
Catalog Number : PCS-31319-PI
Sample ID : 001926C
Description : 0.31mg in 7uL AcOH+55uL MeCN/TFA/H2O DEGASSED

Injection Volume : 3 uL
Data Filename : SYS1-001926C.lcd
Method Filename : gradient 20_80in30min.lcm
Report Filename : reportformat20_80in30min.lcr
Date Acquired : 11/26/2013 7:11:01 PM
Data Processed : 11/26/2013 7:46:34 PM

Gradient: 20-80% in 30 min.
Column: Jupiter 5u, C18, 300A, 547029-24, (4.6mm x 250 mm)
Buffer: A: 0.05% TFA in H2O B: 0.05% TFA in MeCN
Flow: 1mL/min
Instrument ID. SHIMADZU 1

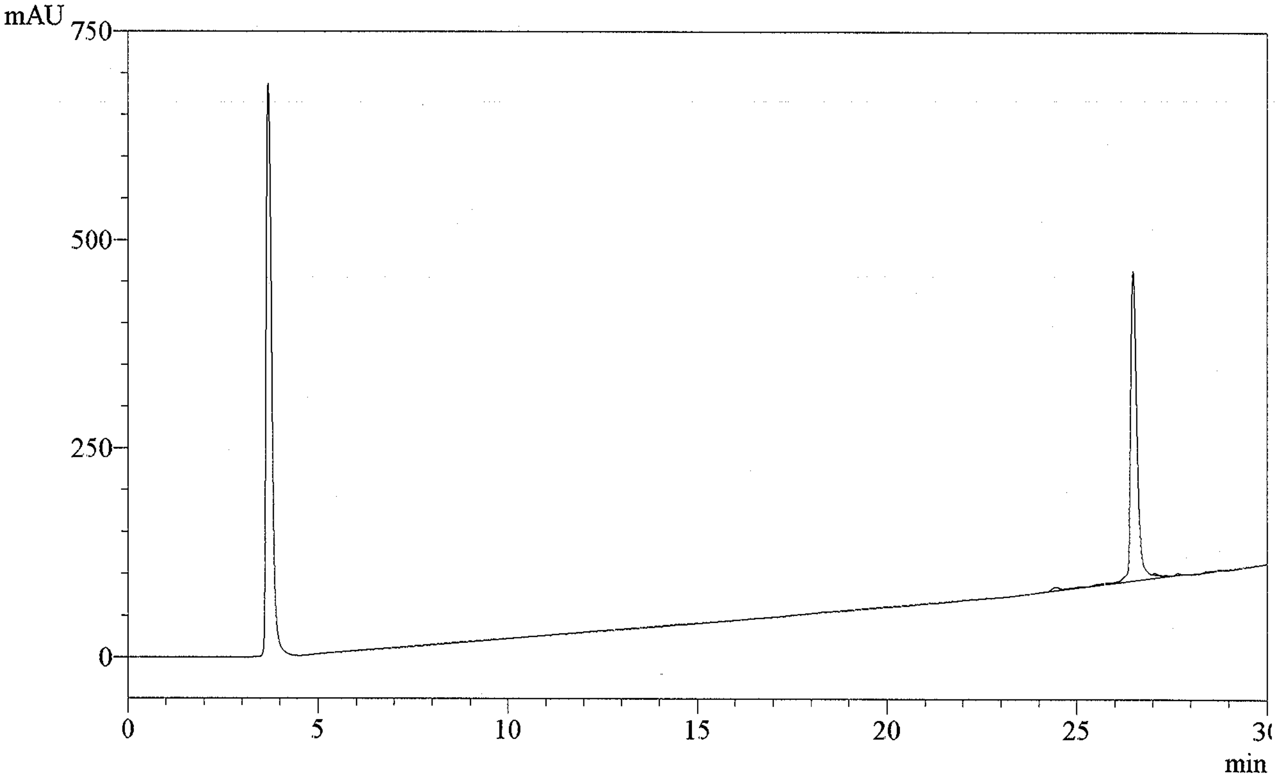

1 Det.A Ch1 / 220nm

PeakTable

Detector A Ch1 220nm

Ret. Time	Area	Height	Area %
24.455	140503	4262	3.060
26.485	4374953	371201	95.284
27.051	11522	1668	0.251
27.340	2947	523	0.064
27.660	16496	2147	0.359
28.125	1030	165	0.022
28.750	44055	2041	0.959
			100.000
