# Supplementary material for: Site-directed M2 proton channel inhibitors enable synergistic combination therapy for rimantadine-resistant pandemic influenza
Source: PLoS Pathog. 2020 Aug 11;16(8):e1008716. doi: 10.1371/journal.ppat.1008716 (PMC7418971; doi:10.1371/journal.ppat.1008716)
Supplement: S1 Table — (DOCX) [file ppat.1008716.s010.docx]

| **Protein** | **Compound** | **Lumenal interactions** | **Peripheral Interactions** |
| --- | --- | --- | --- |
| **N31 (Eng195)** | Rim | **Val27** (x4), **Ala30**, (B,C), **Asn31** (A,D) | Leu40, Ile42 (B), Trp41 (B), Arg45 (B), Asp44, Leu46, Thr43 |
|  | D | **Val27** (A,C,D), **Ala30**, (C,D), **Asn31** (A), Ile33 (C), **Gly34** (D) | Leu40, Ile42 (B), Arg45 (B), Phe47, Asp44, Trp41 (B), Thr43 |
| **S31 (Eng195)** | Rim | His37(x4), **Gly34** (x4), Ile33 (C,D), **Ala30** (D), **Ser31** (A,D) | Phe47, Phe48, Asp44, Leu40, Arg45 (B), Trp41 (B) |
|  | D | His37(x4), **Gly34** (x4), Ile33 (C), **Ser31** (D), **Ala30** (C,D) | Asp44, Phe47, Phe48, Arg45 (B), Leu40, Ile42 (B) |
