# Supplementary material for: Site-directed M2 proton channel inhibitors enable synergistic combination therapy for rimantadine-resistant pandemic influenza
Source: PLoS Pathog. 2020 Aug 11;16(8):e1008716. doi: 10.1371/journal.ppat.1008716 (PMC7418971; doi:10.1371/journal.ppat.1008716)
Supplement: S2 Table — Table summarising the compounds arising from in silico HTS targeting peripheral and/or lumenal binding sites. Compounds were screened for activity in liposome dye release at least twice, with several progressed into toxicity and cell culture studies. Compound names and Chembridge IDs are shown, along with molecular structures and predicted binding (L: Lumen; P: Periphery) based upon E-Hits/Sprout programmes. M2 activity in the presence of compounds (40 μM) is shown for M2-N31/S31 peptides corresponding to the CD or TM regions of the protein. Observed site preferences (Obs L/P) are based upon relative inhibition of M2 CD/TM activity, with “?” indicating potential binding to lumen or partial peripheral binding site based upon compound titrations; bold text indicates differences from predicted binding. Several compounds were tested versus Eng195 in culture (80 μM) and the order of magnitude titre reduction across at least three assays is shown. Finally, IC50 was determined for four compounds selected for synergy experiments. (DOCX) [file ppat.1008716.s011.docx]

| Name,  CB ID | Structure | Pred L / P | *%* M2 activity (40 µM) | | |  | Virus culture | |
| --- | --- | --- | --- | --- | --- | --- | --- | --- |
|  |  |  | N31 CD | N31 TM | S31 CD | **Obs L / P** | Fold Titre Reduction (80 µM) | IC_50_ (µM) |
| Compounds with antiviral activity vs. pH1N1 in culture | | | | | | | | |
| **M2WJ332,**  **-** | 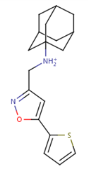 | L | 58.4 | 100 | 85 | **P** | 100x | 1.183 |
| **L1.1, 6607593** | 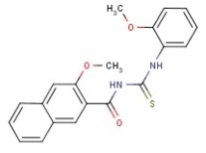 | L | 32.8 | 22.0 | 41.8 | L | 1000x | 1.325 |
| **DL7, 5712740** | 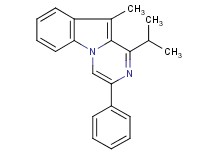 | L | 19.5 | 15.0 | 31.3 | **L/P?** | 100x | 1.482 |
| **DP9, 5107631** | 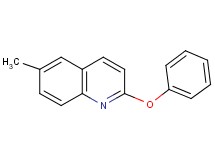 | P | 65.8 | 100 | 97.4 | P | 100x | > 25 |
| **L1, 6622092** | 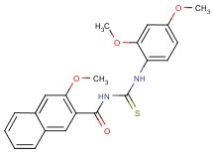 | L | 24.4 | 21.7 | 45.6 | L | 10x | - |
| **L4,**  **7636415** | 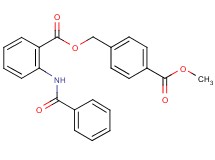 | L | 42.4 | 44.0 | 50.7 | L | 10x | - |
| **P6, 6424106** | 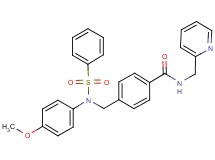 | P | 73.0 | 100 | 75.6 | P | 100x | - |
| **L1.2, 6598617** | 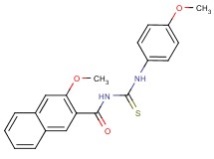 | L | 26.3 | 18.2 | 19.9 | L | 100x | - |
| **L4.2, 7999011** | 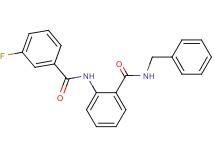 | L | 62.6 | 68.2 | 60.1 | L | 10x | - |
| **P6.4, 5861836** | 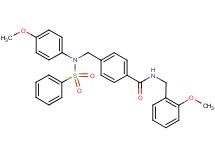 | P | 65.4 | 32.6 | 66.3 | **TM** | 10x | - |
| **DL1, 5315243** | 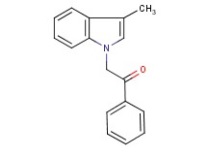 | L | 72.2 | 75.2 | - | L | 0 | - |
| **DL11, 7640329** | 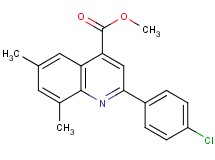 | L | 35.0 | 16.2 | - | L | 10x | - |
| **DP14, 5108039** | 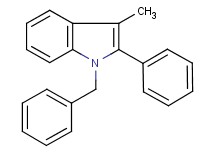 | P | 32.7 | 16.7 | - | **L** | 0 | - |
| **L6, 5525030** | 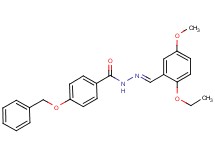 | L | 26.2 | - | - | ND | 10x | - |
| **L7, 7774661** | 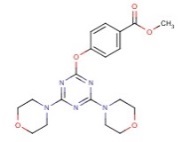 | L | 95.0 | - | - | ND | 10x | - |
| **P1, 7267124** | 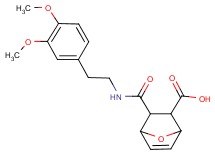 | P | 52.9 | - | - | ND | 10x | - |
| **P4, 7612059** | 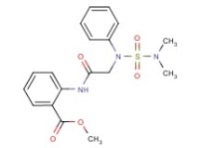 | P | 25.3 | - | - | ND | 0 | - |
| Compounds excluded from cell culture assays | | | | | | | | |
| **DL3, 5631260** | 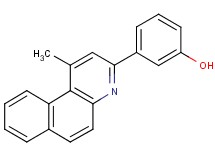 | L | 78.7 | 50.5 | - | L | - | - |
| **L1.3, 6945470** | 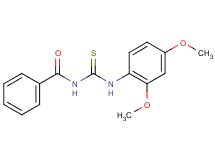 | L | 61.8 | 85.4 | - | L | - | - |
| **L1.4, 7642465** | 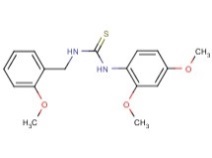 | L | 77.8 | 84.4 | - | L | - | - |
| **L4.4,**  **7749703** | 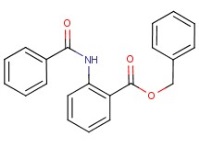 | L | 51.0 | 69.3 | - | L | - | - |
| **DP6, 5120994** | 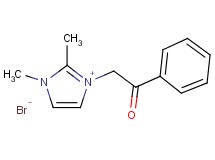 | P | 95.0 | 85.6 | - | **L** | - | - |
| **DP8, 7547759** | 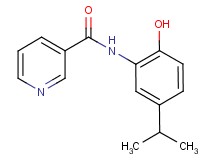 | P | 84.8 | 95.0 | - | **P/L?** | - | - |
| **DP16, 5302843** | 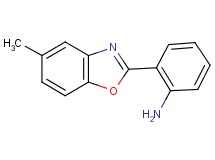 | P | 87.2 | 87.1 | - | **L** | - | - |
| Compounds with activity preference vs TM peptides (note, P6.4 included above with compounds active in virus culture) | | | | | | | | |
| **DL2, 5923305** | 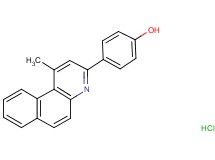 | L | >100 | 72.8 | - | TM | - | - |
| **DL12, 7229212** | 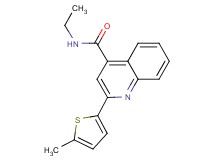 | L | 93.4 | 69.0 | - | TM | - | - |
| **DL4, 9033942** | 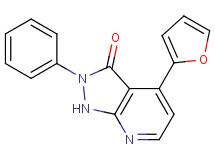 | L | >100 | 74.1 | - | TM | - | - |
| **DL6, 9119119** | 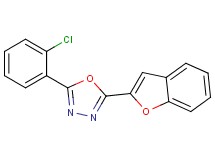 | L | >100 | 51.8 | - | TM | - | - |
| **DP2, 30091343** | 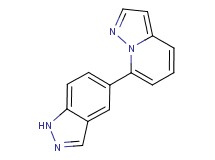 | P | 98.8 | 70.0 | - | TM | - | - |
| **DP3, 21992432** | 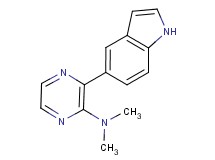 | P | >100 | 63.9 | - | TM | - | - |
| **DP15, 9059508** | 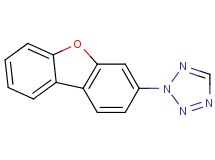 | P | >100 | 70.0 | - | TM | - | - |
| **DP17, 9207910** | 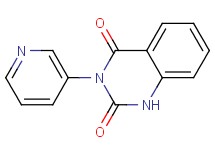 | P | >100 | 86.7 | - | TM | - | - |
| Compounds with negligible anti-M2 activity | | | | | | | | |
| **DP1, 6449207** | 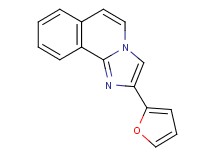 | P | >100 | 90.6 | - |  | - | - |
| **DP4, 7916732** | 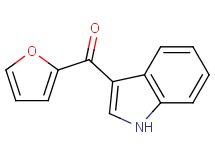 | P | 93.4 | >100 | - |  | - | - |
| **DP5, 5141622** | 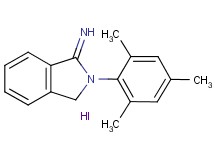 | P | 90.4 | 90.5 | - |  | - | - |
| **P6.2, 6470103** | 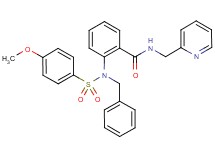 | P | 93.8 | >100 | - |  | - | - |
| **DP7, 5121141** | 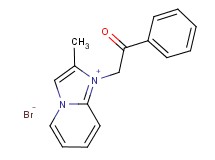 | P | >100 | 97.7 | - |  | - | - |
| **DP12, 7750337** | 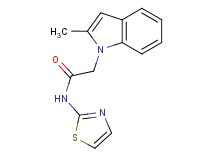 | P | >100 | 91.6 | - |  | - | - |
| **DP13, 5175182** | 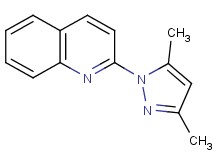 | P | >100 | 92.9 | - |  | - | - |
| **DP19, 5356162** | 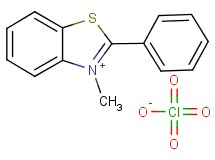 | P | >100 | 95.7 | - |  | - | - |
| **L2, 6553988** | 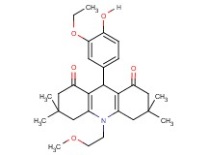 | L | >100 | - | - |  | - | - |
| **L3,**  **5523006** | 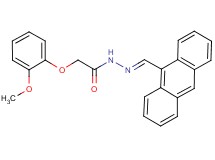 | L | >100 | - | - |  | - | - |
| **L5,**  **5561699** | 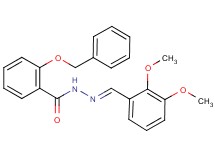 | L | >100 | - | - |  | - | - |
| **P2, 5739630** | 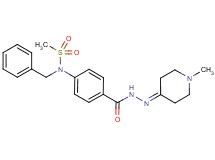 | P | >100 | - | - |  | - | - |
| **P3, 5114273** | 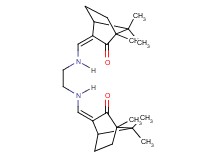 | P | >100 | - | - |  | - | - |
| **P5, 5565578** | 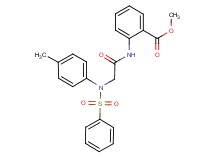 | P | >100 | - | - |  | - | - |
| **L4.3, 5128188** | 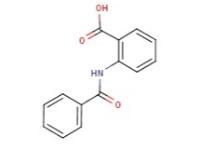 | L | >100 | >100 | - |  | - | - |
| **P6.1, 6479640** | 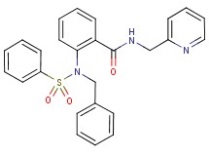 | L | >100 | >100 | - |  | - | - |
| **P6.3, 5732197** | 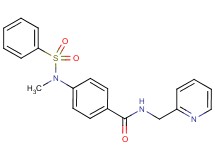 | L | >100 | >100 | - |  | - | - |
| **DL8, 5213091** | 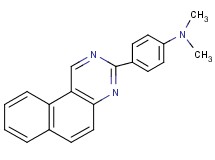 | L | - | - | - |  | - | - |
| **DL9, 5215423** | 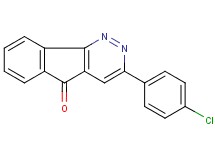 | L | - | - | - |  | - | - |
| **DL10, 5737287** | 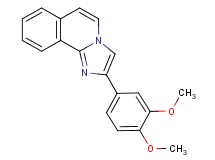 | L | >100 | 99.0 | - |  | - | - |
| **DL13, 6631391** | 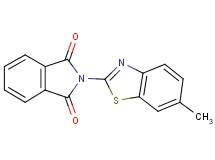 | L | - | - | - |  | - | - |
| **DL14, 5401629** | 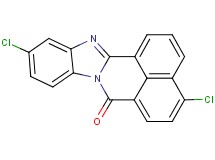 | L | - | - | - |  | - | - |
| **DL15, 6872928** | 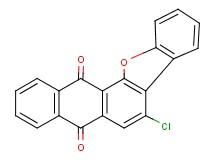 | L | - | - | - |  | - | - |
| **DL16, 7361170** | 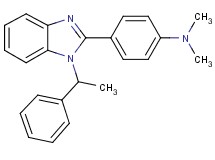 | L | - | - | - |  | - | - |
| **DL17, 6690543** | 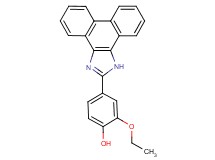 | L | >100 | >100 | - |  | - | - |
| **L4.1, 6302982** | 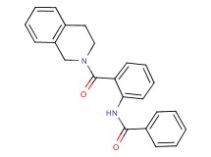 | L | >100 | >100 | - |  | - | - |
| **DL5, 5676101** | 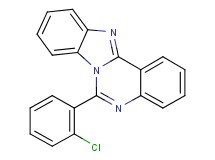 | L | - | - | - |  | - | - |
| **DP18, 5760804** | 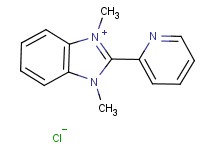 | P | >100 | >100 | - |  | - | - |
| **DP10, 65195788** | 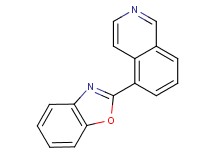 | P | >100 | >100 | - |  | - | - |
| **DP11, 78296026** | 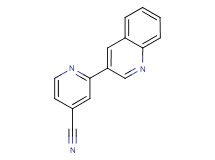 | P | - | - | - |  | - | - |
