# Supplementary material for: Site-directed M2 proton channel inhibitors enable synergistic combination therapy for rimantadine-resistant pandemic influenza
Source: PLoS Pathog. 2020 Aug 11;16(8):e1008716. doi: 10.1371/journal.ppat.1008716 (PMC7418971; doi:10.1371/journal.ppat.1008716)
Supplement: Attachment — Submitted filename: Plos Pathogen Review R1.pdf [file ppat.1008716.s014.pdf]

This revised manuscript by Griffin et al provides additional explanation that the newly discovered M2 channel blockers DP9 and DL7 target the allosteric site at the C-terminal domain, while compound L1.1 targets the N-terminal lumen. During the revision, the authors also provided additional experimental results showing that compound M2WJ332 inhibits proton conductance through the M2-N31 (CD) construct in lipid bilayer. It was suggested that a previously well-characterized compound M2WJ332 targets the C-terminal allosteric site, which contradicts conclusions drawn from previous solution and solid-state NMR results.

It was noted that majority of the issues raised from the previous round of review were not properly addressed. The authors provided no additional experiments such as solution NMR as suggested to support the proposed mechanism of action. The authors also failed to test the three compounds against additional influenza A strains to confirm the mechanism of action and antiviral activity. Therefore, the revised manuscript is still premature for publication.

Basically, there is no solid experimental evidence to suggest that the newly identified compounds DL7, DP9, and L1.1 are indeed M2 channel blockers, and the proposed mechanism of action that DP9 and DL7 target the C-terminal allosteric site was also not experimentally supported. Although the scientific community welcomes controversy, the conclusion needs to be supported by unambiguous experimental data but this study lacks solid experimental results. Here are the main points the authors need to consider:

1) The authors proposed that compound M2WJ332 binds to the C-terminal allosteric site based on the liposome flux assay results. This is the only piece of experimental result the authors provided to support this claim. If the authors have done their literature review thoroughly, they should aware that the binding site of M2WJ332 is well characterized by both solution NMR and solid-state NMR. The N-terminal lumen binding mechanism was also supported by resistance selection. Do the authors believe that liposome flux assay is more reliable than the NOEs from NMR? Second, if M2WJ332 indeed binds to the C-terminal allosteric site as the authors claimed, how could the authors explain that the N-terminal lumen V27A mutant causes drug resistance? The V27A mutant was selected from earlier studies as well as the authors' own study, but the authors seem to skip the explanation.

2) The newly provided electrophysiology data is Fig. S3 did not support the proposed mechanism of action. It only showed that M2WJ332 inhibited current conductance through the M2-N31 (CD) construct. No conclusion can be drawn from this result whether M2WJ332 binds to the N-terminal lumen site or the C-terminal allosteric site. How come the authors did not test the three other compounds DL7, DP9, and L1.1 in this assay?

3) The antiviral activity of the three compounds DL7, DP9, and L1.1 was only tested in one influenza A strain Eng195 which is not sufficient to prove the mechanism of action and the antiviral activity. The authors should at least test these three compounds against one amantadine sensitive influenza A strain (M2 -S31). If the proposed mechanism of action is correct, luminal-binding compound L1.1 should not be active, while C-terminal allosteric inhibitors DP9 and DL7 should be active.

4) The fact that the authors failed to select resistant mutants for DL7 is a strong evidence that DL7 is not a M2 channel blocker. This evidence argues against the authors' own statement.

5) “Remarkably, combinations of M2WJ332 with either L1.1 or DP9 yielded synergistic reductions of viral titre (Figure 7a).”

Comment: if M2WJ332 and DP9 bind to the same luminal site, how could they achieve synergistic effect? Any logic?

6) Figure S5. Metabolic activity of M2-N31 inhibitor treated cells.

Comment: this should not be called metabolic activity, it is cellular cytotoxicity
