# Supplementary material for: Site-directed M2 proton channel inhibitors enable synergistic combination therapy for rimantadine-resistant pandemic influenza
Source: PLoS Pathog. 2020 Aug 11;16(8):e1008716. doi: 10.1371/journal.ppat.1008716 (PMC7418971; doi:10.1371/journal.ppat.1008716)
Supplement: Attachment — Submitted filename: SG-responsetoreviewsPLoSPath2020v2.docx [file ppat.1008716.s013.docx]

**Dr Stephen Griffin**

**Associate Professor**

**Academic Lead for Laboratories**

School of Medicine

University of Leeds

St James’ University Hospital

Leeds, LS9 7TF

Tel: (+44)113 3438637

E-mail: [s.d.c.griffin@leeds.ac.uk](mailto:s.d.c.griffin@leeds.ac.uk)	27^th^ April, 2020

Dear Profs Lowen, Pekosz, Haldar and McFadden

Thank you for providing the reviewer feedback on our manuscript *“Site-directed proton channel inhibitors as synergistic combination therapy for rimantadine-resistant pandemic influenza”.* We were delighted with the positive views of reviewer two and Prof Busath, as well as the many constructive points raised by reviewer one. We are happy to provide detailed explanation of the steps we have taken to improve the manuscript, including the provision of additional data and a considered rebuttal where appropriate. Notably, we confirm that the M2WJ332 compound displays a preference for M2-N31 conductance domain peptides using an orthogonal ion channel assay.

We consider that the accommodation of the reviews has led to a much-improved manuscript, which we hope will be acceptable for publication in *PLoS Pathogens*.

Please do not hesitate to contact me if any points are unclear, or for any additional information. I look forward to hearing from you in due course.

Yours Sincerely

Dr Stephen Griffin, MA(Hons) Cantab, PhD Cantab

Part 1, Summary:

Reviewer 1.

Strength:
1) it raised a scientific controversy which will attract significant attention from the scientific community of M2.

2) The resistance selection experiment is well executed and the results make sense.

We thank the reviewer for acknowledging that the area concerning potential inhibitor binding sites on the M2 channel complex is worthy of further exploration.

Weakness:
1) The conclusion was not supported by stringent experimental data.

All experiments presented herein are, in our view, appropriately controlled and neither reviewer 2, nor reviewer 3 have stated to the contrary. In the absence of a specific criticism, we find it difficult to address this point directly here, and refer the reviewer to the responses in the “major issues” section.

2) It is important to include at least two independent assays to validate the proposed mechanism of action. In this study, the only experiment conducted was the liposome flux assay.

We acknowledge that the dye release assay is an indirect measure of M2 proton channel activity. Nevertheless, the system recapitulates M2 channel activation at acidic pH and can discriminate between rimantadine sensitive/resistant S/N31 channel activities. Moreover, screening using this method has far greater processivity compared to electrophysiological assays and results in the identification of multiple biologically active compounds with antiviral activity. We have observed similar outcomes for other viroporins (e.g. HCV p7, HPV16 E5) and Boehringer Ingelheim validated the system as a robust HTS with reliable Z scores (PMID: 21343600).

The dye release assay has enabled the first direct comparison of CD and TM peptide inhibitor sensitivity in a functional context where their activities were equivalent; reviewer 3 notes this as a strength. Whether our interpretation of this corresponding to the peripheral/lumenal targeting of compounds is correct or not (see below), this suggests that conclusions based on the study of only one or other peptide cannot necessarily be related to the full-length protein; both are needed. As discussed at length in the manuscript, structural studies carry bias due to the differences in the *trans*-membrane bundles formed using one or other peptide, and this has implications for drug-bound structures derived at high (usually millimolar range) compound concentrations. The flaw in confirming one site as genuine using a system lacking the second site should not need elaboration.

However, given the concerns of this reviewer and other comments, we have now included an orthogonal electrophysiological assessment of the ability of M2WJ332 to inhibit M2-N31 CD and TM peptides (see new figure 3E, 3F and supplementary figure S3). Whilst higher concentrations of the compound (2000 nM) show a modest effect vs TM peptide in this system, low (400 nM) abrogate activity of CD peptides. Thus, we conclude that liposome dye release assays confer an accurate representation of inhibitor targeting versus the two peptides.

Reviewer #2: More than 95% of the currently circulating influenza A viruses carry the amantadine and rimantadine-resistant M2-S31N proton channel. For this reason the topic of the current manuscript, the study of the M2-S31N channel and the design of M2-S31N inhibitors taking into account the controversy surrounding the two potential drug binding sites for Amt, is of the greatest interest.

The overall quality of the manuscript is excellent. The paper is concise, very well written and the experimental design is straightforward. The introduction is clear and up-to-date. Both the computational and the biology parts of the work have been skillfully conducted and the results are clearly presented and discussed.

We thank the reviewer for these positive comments.

Reviewer #3: Scott et al report extensive work leading to the observation of a novel anti-influenza compound, L1.1, two additional compounds of possible value, DL7 and DP9, and the discovery of resistance formation to a previous invention M2WJ332 via M2 V27A.

The means of identifying the novel compounds are dubious: in silico screening of two established M2 binding sites, the accepted lumenal amantadine binding site and a peripheral binding site in the micellar M2 CD structure that is largely abrogated in the membrane protein structure, and carboxyfluorescein release from M2-proteoliposomes, which has not been calibrated against electrophysiology and depends on the assumption that a bulky dye is somehow transported by the extremely narrow M2 channel.

We agree that our choice of docking template was not ideal, as discussed in the manuscript and explored in figure S8 (note, new numbering); Ideally we would have used the membrane structure from the Cross laboratory, although this may also suffer some limitations as it is not a drug-bound structure. We suspect that this explains the relatively high numbers of successful lumenally-, versus peripherally-, targeted compounds.

The hydrodynamic (Stokes’) radius of Carboxyfluorescein is approximately 0.8 nm. The reviewer rightly notes that the constrictions at His37 and Trp41 are much narrower, yet various static and molecular dynamics simulations estimate the wider regions of the M2 pore to measure 0.6 nm or more. Given that acidic pH increases CF transport in the assay, we presume that passage of CF at neutral pH may also be dependent upon a dynamic equilibrium involving His37 and opening of the channel structure.

Nevertheless, the dye release assay has been used extensively by the authors for assessment of the broader (OuYang et al, 2013, Nature 498:521-525) P7 channels in hepatitis C, gave internally consistent results (M2WJ332 blocked the N31 M2 transport and not the S31 M2 transport, as found in electrophysiology), and most importantly led to the discovery of compounds with outstanding potency against influenza infections in cell culture.

The reviewer rightly points out that we have also employed dye release assays to identify biologically active small molecule inhibitors of p7 and other viroporins, as discussed briefly above. With respect to lumen size and the dye release assay, we point out that the Ouyang p7 structure is controversial (Oestringer *et al*., Nature 2019) and that we and others have generated NMR-based models with much narrower apertures (Foster *et al*., Hepatology 2014; Scull *et al*., PLoS Path 2015). Moreover, the latter are consistent with biochemically defined size limits for p7 conductivity (StGelais *et al*., Antiviral Research, 2007).

Furthermore, the proteoliposome approach allowed easy comparison of dye release properties of M2 TMD and M2 CD, which is novel. However, the authors may have been too accepting of the assumption that compounds that reduce dye release in the CD but not the TMD must be blocking peripherally, as this assumption has not been independently validated.

As discussed above (reviewer 1), we have now included orthogonal validation of M2WJ332 binding preference for CD peptides (see new figure 3E, 3F and supplementary figure S3). However, we accept the point that other possibilities might account for differences in drug sensitivity. Thus, we have moderated language in the discussion with direct reference to one or other site, and we also discuss alternative explanations; indeed, the TM-specific compounds suggest that peptide conformations as well as additional binding sites may play a significant role.

The study and article are very well-crafted and references the current literature accurately and thoroughly. The scientific methodology is similarly state-of-art and thorough.

We thank the reviewer for this supportive comment.

The only general concern is that perhaps the results are slightly over-interpreted. Carboxyfluorescein transport may be mediated by M2 monomer insertions, given that the peptide is added to the dye-containing vesicles post hoc.

We accept that M2 monomers being responsible for CF release is a possibility, yet we consider it a remote one for the following reasons:

1. It is difficult to reconcile how small molecule inhibitors might prevent monomer-induced release in a manner dependent upon the presence of either N or S at position 31 (M2, rather than peptide sequence), or indeed for a shorter versus a longer peptide.

2. Analysis of the imino-sugar *N*N-DNJ demonstrated that disruption of oligomer formation correlated with channel inhibition (Figure S1). Moreover, it was necessary to use highly denaturing conditions (Urea and SDS) combined with chemical cross-linkers to resolve M2 monomers and lower oligomeric forms versus tetramers as native PAGE and other techniques only resolved oligomers.

3. The newly added electrophysiology experiments confirm that M2 peptides form single channels (figure S3). Furthermore, addition of M2-N31 CD peptides increases the normalised conductance of lipid bilayer by 3-4 orders, yet conductance returned to the level comparable to that of a black lipid bilayer following addition of M2WJ332. Again, this supports the insertion of M2 peptides into the lipid bilayer to form drug sensitive channels; inhibitors are unlikely to be able to repair a disrupted bilayer or bind to monomers.

The designations of peripheral and luminal binding based on dye release may be based on false assumptions.

As above, we have moderated the language used to describe our findings in relation to this point in the discussion.

The DL7-resistant virus should have shown mutations too.

We agree. Please see response in minor issues section below.

L1.1 seems to be too large to get past the V27 barrier and get into the luminal site in M2. The lack of resistance developing in the presence of L1.1 could mean that it’s blocking a physiologically critical site in M2; or, just that L1.1 is blocking some other key viral or host process, and that the dye release result is a red herring. Likewise for the lack of M2 sequence change in 9/9 sequences of plaque-purified L1.1-passaged virus.

It is correct that the M2 lumen has narrow constrictions in the NMR structure, as well as other crystal structures. However, whilst L1.1 is theoretically too large to access this region in the fixed structure, it is no larger than many of the compounds thought to bind within the lumen; its molecular diameter is smaller than rimantadine, for example. It is the case that N31 causes a widening of the lumenal aperture compared to S31 containing channels, which was attributed to allowing M2WJ332 binding within the lumen of peptides derived from H3N2 Udorn M2 with an S31N mutation (Wang et al., PNAS 2012).

Much like the discussion of M2 carboxyfluorescein transport, what the fixed structure does not show is the potential for dynamism of the backbone and side-chains. For example, rotation of the Trp41 side chains upon channel opening generates a far wider aperture, and this likely also affects His37 and Val27 constrictions; this is consistent with increased activity at lower pH in the assay, presumably inducing such changes via His37 protonation. It would require extensive molecular dynamics simulations to assess how small molecules might access this region under various conditions that are beyond the scope of this study.

With respect to the selection, or not, of L1.1 resistance, it is difficult to discern between the possibilities raised by the reviewer as this involves interpreting a negative result. In support of our hypothesis that L1.1 does indeed target a critical site, we can only say the following: 1) we know from M2WJ332 selection that the protocol does allow changes in M2 to manifest in response to inhibitor treatment. 2) Unlike DL7 treated virus (see below), L1.1 treated virus evolved non-synonymous mutations under selection in addition to synonymous changes. Please see DL7 response below for details. 3) Unlike DL7, L1.1 is a member of a structurally related series; L1, L1.1 and L1.2 all display antiviral activity whereas L1.3 and L1.4 do not. Consistently, L1.1 and L1.2 are modified (red circles, below) in a different region to L1.3 and L1.4, supporting the importance of the latter region for M2 targeting, although this can’t be confirmed without more extensive testing.

**Part II – Major Issues: Key Experiments Required for Acceptance**
Please use this section to detail the key new experiments or modifications of existing experiments that should be absolutely required to validate study conclusions.

Generally, there should be no more than 3 such required experiments or major modifications for a "Major Revision" recommendation. If more than 3 experiments are necessary to validate the study conclusions, then you are encouraged to recommend "Reject".


Reviewer #1: This study by Griffin et al. is interesting. It brought a new scientific controversy regarding the drug binding site(s) of the influenza A virus M2 proton channel.

In this study, the authors used liposome flux assays to screen non-adamantane based inhibitors for the M2-S31N mutant, which is the predominant drug resistant mutant in circulating influenza A viruses. In this assay, they incorporated two M2 constructs, the transmembrane domain (TM) which only contains the luminal drug-binding site, and the conductance domain (CD) which contains both the luminal drug-binding site and the peripheral drug- binding site.

There was also the S31 CD peptide, which allowed validation of the assay.

They first attempted to prove the validity/feasibility of the liposome flux assay using known M2 channel blocker rimantadine plus a few other compounds that may or may not be relevant.

The M2 inhibitor literature comprises multiple studies utilising diverse adamantane derivatives. This includes those preceding the identification of M2WJ332 and related compounds, the origins of which began with the fusion of a spirane moiety to amantadine. Next followed numerous examples of heterocycles used as “warheads” to add to amantadine. We utilised the compounds in figure 1 as we had previously show some of them were capable of inhibiting rimantadine resistant HCV p7 channels (Foster *et al*., Hepatology 2011).

Super singly, they claimed that a previously well-characterized compound WJ332 actually inhibited M2 through targeting the peripheral site, not the luminal site. Nevertheless, the authors then conducted a virtual screening to identify novel compounds that can bind to either the luminal site or the peripheral site or both. Four non-adamantane compounds were identified and further confirmed in the liposome flux assay.

We refer the reviewer to table 1, which details over 30 novel (out of 62 tested) inhibitors of M2 channel activity *in vitro*. We feel this high percentage hit rate supports that our *in silico* enrichment for each target based upon molecular fit was a success.

The antiviral activity of two compounds L1.1 and DL7 were further confirmed in viral replication assay.

Seventeen compounds, along with M2WJ332, were tested in virus plaque reduction assays. We focused upon L1.1, DL7 and DP9 as these represented the best hits with alternative predicted target specificities.

Next, the authors performed resistance selection experiments and found no M2 mutations for L1.1, but V27A mutant for WJ332. Finally, compound L1.1 was shown to have synergistic antiviral activity with either WJ332 or zanamivir.

Although the authors claimed interesting and somewhat controversy findings, the conclusions are not supported by the experiment results. Many of the experiments are poorly designed and lacks vigorous controls.

Again, we would be grateful if the reviewer might state which controls are missing, and from which assays.

Major comments are:
1) The accuracy and reliability of the liposome flux assay is in doubt. There is no stringent calibration/validation for this assay. For example:

As aforementioned, we believe this assay strikes a favourable balance between processivity and indirect measurement of channel activity/inhibitor effects. We are aware of its limitations, which is why it represents a first pass biological screen, supported by subsequent virological assays and other methods.

a. In Fig. 1B, rimantadine only inhibited 50% of channel activity of M2-WT (31) at concentrations up to 40 uM. This is in direct contrast to the efficacy of rimantadine. Rimantadine inhibits M2-WT containing viruses with submicromolar efficacy and blocks M2-WT channel with single micromolar efficacy in electrophysiological assays.

We have claimed at no point that EC_50_ values derived in the liposome assay are comparable to other systems, and would not do so. It is common practice in drug discovery to utilise *in vitro* screening assays as a first pass filter to screen extensive compound libraries – indeed, such assays often involve highly sensitive direct binding and/or disruption of enzymatic function, which can generate very low hit EC_50_ values. By contrast, the dye release assay requires relatively high concentrations of otherwise (in other systems) potent inhibitors in order to register a “hit”; this has the effect of filtering out noise in the system as low potency hits require yet higher concentrations to register.

Notably, M2WJ332 also has a similar EC_50_ in liposomes compared to rimantadine, and yet shows nanomolar potency in suspended bilayer assays and low micromolar potency in E195 virus culture. We neither believe that it is necessary, nor accurate to extrapolate EC_50_ values from one assay to another in order to derive potent antivirals. In support of this, our combined *in silico* and *in vitro* screening approach generated an enriched hit rate of compounds with genuine antiviral potency, despite the high bar set by the dye release assay to register activity.

b. In Fig. 1B, compounds D, H, J were shown to potently inhibit both M2-WT and M2-S31N in the liposome flux assay; therefore, these three compounds should inhibit both M2-WT and M2-S31N containing viruses. However, no such data was provided. Similarly, antiviral assays should be performed for compounds B and K to test whether they only inhibit M2-WT containing viruses, but not M2-S31N containing viruses.

Apologies, we now include the data for H, J and NNDNJ, all of which displayed antiviral activity versus E195 (new figure S2). However, these were not tested versus a rimantadine sensitive virus.

We revisited B and K as this is an excellent suggestion. Unfortunately, as was the case for several *in vitro* hits, neither B nor K exhibited antiviral effects in cells against either E195, or E195 engineered to express M2-S31; the L1.1 positive control worked in all cases. Note, we did not include this experiment or reference it in the revised text as we felt it disrupted the flow of the manuscript, as L1.1 is not described until later figures. Instead, it is shown here (MDCK plaque reduction assay, 24 hr post-infection, inhibitors at 80 micromolar):

c. In Fig. 3C, WJ332 only inhibited ~40% of channel activity of the N31 CD peptide in the liposome flux assay when tested at up to 40 uM. This is in direct contrast to the efficacy of WJ332 in electrophysiological assay and antiviral plaque assay. WJ332 had an IC50 of 16 uM in two-electrode voltage clamp assay and an EC50 of 153 nM in antiviral plaque assay (PNAS January 22, 2013 110 (4) 1315-1320).

We refer the reviewer to the response above concerning rimantadine. We also do not agree that it is accurate to compare compound efficacies between studies, unless one is deliberately attempting to recapitulate the exact experimental conditions of the other.

However, we note that the values quoted above for M2WJ332 in the two systems are an order of magnitude apart, reminiscent of dye release assays for M2WJ332 versus plaque reduction assays herein. M2WJ332 activity in the dye release assay is within the same order of magnitude as the voltage clamp assay described above, whereas the cell culture efficacy differs by approximately 6/7-fold.

Notwithstanding differences between assay systems and cell culture protocols, the studies cited above utilised protein/peptides from the amantadine sensitive H3N2 Udorn strain with an engineered S31N mutation combined with naturally resistant H1N1 WSN virus, isolated in 1933. We used the same authentic H1N1 E195 resistant strain from 2009 for all assays. Sequence differences could therefore also account for differences in inhibitor efficacy.

d. Both the N31 CD and N31 TM peptides contain the luminal drug-binding site, which means compounds that bind to the luminal site should show equal or more potent channel inhibition for the N31 CD construct than the N31 TM construct (for example, compounds that happen to bind to both luminal and peripheral sites). However, this is not the case for compound P6.1 (Fig. 4B), there must be something seriously wrong with this assay.

We apologise, but we do not understand the reviewer’s logic. Why would lumenally targeting compounds show greater effects versus a CD peptide compared to a TM? We are uncertain how to answer this query. With reference to compounds binding two sites, we emphasise that this reflects ambiguous docking caused by equivalently poor molecular fit within each site, rather than the compounds necessarily binding in two places. Exploring preferences for one or other site and ideally identifying compounds that target one or other was the defining rationale for this study.

We assume that the reviewer is referring to the TM-targeted compound 6.4, rather than 6.1. In which case, this compound is not alone in adopting this behaviour (see table 1). However, 6.4 was the only member of this compound class to display any sign of (weak) antiviral activity. We therefore consider such compounds may show selectivity versus a more open-form channel, as discussed in the manuscript. This form does not appear to be biologically relevant, but supports that peptide conformations likely influence binding, at least *in vitro*, as our newly modified discussion now reflects.

2) The claim that WJ332 is a peripheral M2 inhibitor is premature. The ONLY piece of experimental data the author provided to support this claim is in Fig. 3C, in which WJ332 appeared to only slightly inhibit N31 CD but not N31 TM. To confirm/validate a proposed drug binding mechanism, here are the general approaches:

As described above, we have now confirmed this observation using an orthogonal system. We thank the reviewer for their personal insight into mechanistic validation.

1) Determine the drug-protein bound structure. The structure of WJ332 in complex of M2-S31N was solved by solution NMR (PDB: 2LY0), which showed luminal drug binding. In addition, the drug binding of similar compounds was determined by either solution NMR or solid state NMR (J Am Chem Soc 135, 9885-9897; J. Am. Chem. Soc. 136, 17987-13995).

As discussed on several occasions, structures based upon either TM or CD peptides carry inherent bias, which in turn depends upon the detergent or lipidic environment in which they were derived. Notably, other well-regarded studies of M2 peptides in bilayers have confirmed that both lumenal and peripheral sites are indeed present upon M2 channel complexes in lipid bilayers (Cady *et al*., Nature 2010; Rosenberg & Casarotto, PNAS 2010).

The studies mentioned by the reviewer are based upon TM peptides from a prototypic strain, engineered to contain the S31N mutation. Thus, they lack the majority of the peripheral site making comparative binding analysis impossible, in our view.

2) Mutagenesis. Mutations on the residues located at the proposed drug-binding site should confer drug resistance in both electrophysiological assay as well as cellular antiviral assay. Mutations (V27A, N31S, N31D, I32T) located at the luminal drug-binding site have been shown to confer resistance against M2WJ332 and similar compounds (Mol. Pharmacol. 2016, mol.116.105346; Antiviral Res. 2018, 153, 10-22.). In this study, the authors also selected V27A mutant with the drug selection pressure of WJ332, which supports the luminal binding mode.

The mechanism underpinning M2 resistance to rimantadine and related compounds is confounded by the fact that many of the mutations listed above (and other rarer variants) are known rimantadine resistance mutations However, they are not in fact proximal to the lumenal position of rimantadine or amantadine seen in the majority of atomic structures. The fact that these positions do interact with lumenally bound M2WJ332 in PDB: 2LY0 is a consequence of its inverted orientation and longer sidechain.

Naturally, V27A would disrupt interactions with M2WJ332 in the 2LY0 structure and in our own docking predictions. However, firstly, this mutation removes a minority of the hydrophobic interactions stabilising the adamantyl cage of M2WJ332 within the helical bundle, leaving a large number of polar interactions and H-bonding intact. Second, as discussed in our manuscript, Pielak *et al*., described an additional mechanism of action for mutations at positions 27 and 31 via destabilisation of channel complexes (Pielak *et al*., PNAS 2009). Again, the lack of comparative studies make either option potentially viable. We note that D44A mutations also disrupt peripheral inhibitor binding (Rosenberg & Casarotto, PNAS 2009).

3) Structure-activity relationship studies. Numerous analogs of WJ332 have been synthesized and tested, and the SAR is consistent with the luminal drug-binding mode.

We understand that multiple analogues of M2WJ332 have been tested, with variation in the first 5-membered ring (isoxazole-, 1,2,4- oxadiazole–, and isoxazoline-) and multiple variations in the adjacent R-adduct. However, whilst these compounds may well be consistent with lumenal binding, this was again a study where TM peptides and full-length protein/virological assays were used; no comparative measure was taken.

Since a NMR expert, Jason Schnell, was listed as a coauthor, why don’t they solve the solution NMR structure of M2-S31N CD construct in complex with L1.1, DL7, and DP9 if they truly believe these molecules are M2 inhibitors? Maybe they should also repeat the NMR experiment with WJ332 and prove that WJ332 binds to the peripheral site but not the luminal site! It appears the scientific controversy never settles, the first one is related to amantadine binding to M2 WT, now it comes to WJ332/L1.1 binding to M2 S31N. This is exciting!

Solving such structures might result in biased outcomes as described above. The most meaningful structures would be those of full length M2 within lipid bilayers, yet this remains elusive. Thus, solving eight NMR structures (TM/CD unbound and with the three inhibitors) is not only outside the scope of this investigation, it is likely not achievable within a meaningful timeframe and would likely prove inconclusive.

3) Following the 2nd point, the author failed to provide any relevant experimental results, other than the questionable liposome flux assay, to support the proposed luminal binding mode or the peripheral binding mode. Will mutations located at either the luminal or the peripheral binding sites of L1.1 confer resistance against this compound in both liposome flux assay and antiviral assay?

We do not consider the liposome assay to be questionable, as discussed above. Accordingly, we have now confirmed M2WJ332 preferences for CD peptides in an orthogonal system. L1.1 does not have a predicted interaction at the peripheral site and we did not select resistance mutations to this compound. We are therefore uncertain how to answer this question.

4) No M2 mutant was found during the serial viral passage experiment with L1.1, which means this compound inhibit influenza A virus through a different mechanism of action other than targeting M2 directly!

Please see responses to reviewer three (Prof Busath).

5) M2 is a proton channel and almost all of the potent M2 channel blockers contain a positively charged amino group or equivalent, which serves as a mimetic of the conducting hydronium ion. However, all three hits identified in this study L1.1, DL7, and DP9 are neutral compounds. The proposed binding modes shown in Fig. S2 do not seem to make sense. The interactions appeared to be mainly driven by hydrophobic interactions.

We accept that most adamantane-based inhibitors of M2 identified to date possess these characteristics. Our inhibitors represent distinct chemotypes and so are unlikely to interact with the lumen with the same binding mode. This notion is supported by our docking predictions (figure S4).

6) Compound L1.1 should also be tested against other influenza A and B viruses to make sure it does not have non-specific antiviral activity. WJ332 and related compounds have been extensively tested against multiple influenza A and B viruses.

We have now shown that L1.1 is also active against ENG195 containing an N31S mutation, consistent with *in vitro* data (see above). Further testing was interrupted due to the COVID19 lockdown. We understand the reasoning behind this request, but also point out that pandemic 2009 H1N1 is one of the major circulating strains at present, and so is more clinically relevant compared to various historical/laboratory strains.

7) In the combination therapy experiment, how could two compound that compete for the same drug-binding site have synergistic effect? This makes no sense.

We assume that the reviewer refers to the combination of M2WJ332 and DP9. We attribute this to the presence of up to four potential binding sites upon the M2 channel periphery. Accordingly, mild antagonism occurred when higher concentrations of M2WJ332 are present, suggesting a switch from a co-operative to a competitive process as these sites become increasingly saturated. We have added text to the discussion to explain this hypothesis.

8) How could L1.1 be classified as a luminal binding compound? There is no difference in N31 CD and N31 TM inhibition as shown in Fig. 4B.

We apologise if our reasoning is unclear to the reviewer. Whilst both CD and TM peptides contain a lumen, only CD peptides retain the full peripheral site. Thus, we interpret compound activity against both as being attributable to binding within the lumen.

Reviewer #2: (No Response)

Reviewer #3: (No Response)

**Part III – Minor Issues: Editorial and Data Presentation Modifications**
Please use this section for editorial suggestions as well as relatively minor modifications of existing data that would enhance clarity.


Reviewer #1: Minor comments:
1) Introduction: M2 is a 97-residue long peptide, not 96-residue.

M2 is initially translated as a 97 amino acid protein. However, as for the vast majority of proteins in Eukaryotic cells, it is a substrate for methionine aminopeptidases. Hence, the abundant mature protein expressed in cells and, critically, present within virions is 96 amino acids in length, with Ser2 comprising the N-terminus. We have amended the text to state that mature M2 comprises 96 residues.

2) Introduction: it states “ensuing controversy remains”. This is an inappropriate description. Since the two Nature papers published in 2008, numerous studies have been conducted to validate the pharmacology relevant drug binding sites of M2, including Dr. Chou’s own study. I think it is fair to claim that the controversy has settled.

The key message from our study, as we attempted to set out in both the introduction and the discussion, is that both of the amantadine binding sites within the IAV M2 protein are amenable to development via modern drug discovery. Both sites are relevant in studies of the protein within lipid bilayers (Cady *et al*., Nature 2010; Rosenberg & Casarotto, PNAS 2010).

We are naturally aware of the chimaeric influenza A and B M2 studies, as well as mutagenic studies of the peripheral binding site (Chou, Lamb and Pinto laboratories). However, we do not consider these to be conclusive as the removal of one site by mutagenesis will necessarily favour the other, and chimaeric proteins are not relevant in nature. Nevertheless, we concede that “controversy” may be a strong word and have amended the text.

3) Why are compounds L, B, and K called amiloride-related compounds? There is absolutely no structural similarity.

We apologise that this has caused confusion. We meant that K, L, and to a lesser extent B, are structurally related to amilorides such as HMA; we have amended the text accordingly. We acknowledge that these compounds have different substructures, but they retain the same molecular features, i.e. K and L are amino-substituted heterocycles. B is less obviously related in terms of structure, yet contains the same pharmacophore, namely a basic centre and heterocycle, roughly three atoms apart.

4) There is no characterization for the M2 peptides.

We apologise for this oversight. Full synthetic data from Peptides International are now provided as supplementary material.

5) Molecule in Fig. 3D is wrong. Anyone with chemistry background should realize that the molecule shown in Fig. 3D is not the same molecule in Fig. 3B. How could isoxazole be protonated?

We apologise for this oversight. We have replaced the figure in question with images of the correct binding pose/molecule and appropriate protonation.

Please also note that, as requested by editorial staff, we have deleted the reference to M2WJ332 effects at higher concentrations upon TM peptides as they were not performed the same number of times as the titrations up to 40 micromolar.

Reviewer #2: (No Response)

Reviewer #3: Table 1 could be consigned to SI, except for the first four rows, as long as the selection process is better explained in the Table legend.

With the reviewer’s/editor’s permission, we would prefer to keep the table as it stands. Our concern would be that, to some (including reviewer 1), the extent of the screen and the number of hits may not be obvious due to our focus within the manuscript upon key exemplars. Moreover, a single table permits convenient location of all inhibitors employed, allowing comparison of effective vs ineffective structures, and cuts down on the level of cross-referencing within the manuscript.

Figure 6b: What about the others? No change in Rim because, even at 80 uM it is ineffective. No effect of Zan on M2. DMSO (what %?) passaging should not affect M2 very quickly, right? Didn't DL7 produce resistance yet? It has resistance by P14 according to fig 6D..., but apparently it hasn't developed yet at P6 according to fig 6C. What about the DL7 plaques?

Cells were subjected to a maximum of 1 % DMSO. Standard sequencing of Rim and Zan treated cultures showed no detectable changes in the M2 sequence – unfortunately, these were not subjected to whole genome NGS; this was performed on the DMSO control as well as virus selected by L1.1, M2WJ332 and DL7. We also sequenced virus from L1.1 and M2WJ332 selected plaques.

We are similarly confounded by the results with the DL7 compound and accept that the focus of the paper is, as a consequence, upon L1.1 and M2WJ332. We unfortunately did not analyse DL7 plaques. However, upon revisiting our NGS data, it is clear that the DL7 compound does not exert the same degree of influence upon viral evolution as the other two inhibitors. Specifically, whilst the number of synonymous base changes are similar for all conditions, the DL7 selected virus generates only two distinct non-synonymous changes in its sequence over at passages 5 and 14; this is fewer than the number seen in the DMSO control. By contrast, around 20% of mutations induced by both L1.1 and M2WJ332 selection generate non-synonymous changes, including the highly abundant V27A change in the M2 sequence seen for the latter.

We are uncertain how to interpret these data as all other experiments support a direct effect upon M2 and DL7 shows equivalent potency compared with L1.1 and M2WJ332. However, NGS data argues that the virus is under no more selection pressure during multiple passages than compared to the DMSO control. One interpretation is that DL7 has no direct-acting antiviral effect in long-term culture. Thus, the low-level virus titration at passage 6 may be an artefactual consequence of our bulk passage methodology, rectified upon subsequent normalisation of innoculae during passage 7. However, data from plaque reduction and *in vitro* assays contradict this notion. Mutation rates are summarised in the table below, and this has now been included as data.

|  | **Day 5** |  |  | **Day 14** |  |  |  |  |
| --- | --- | --- | --- | --- | --- | --- | --- | --- |
| **Condition** | **Syn** | **Non-Syn** | **Total** | **Syn** | **Non-Syn** | **Total** | **Total Changes** | **% NS** |
| DMSO | 36 | 3 | 39 | 38 | 2 | 40 | 79 | 6.32911392 |
| M2WJ332 | 10 | 3 | 13 | 45 | 12 | 57 | 70 | 21.4285714 |
| M2WJ332 plaque | 11 | 3 | 14 | ND | ND |  | 14 | 21.4285714 |
| L1.1 | 22 | 3 | 25 | 31 | 9 | 40 | 56 | 21.4285714 |
| L1.1 plaque | 16 | 3 | 19 | ND | ND |  | 19 | 15.7894737 |
| DL7 | 29 | 1 | 30 | 32 | 1 | 33 | 63 | 3.17460317 |

Nevertheless, we remain convinced that our system enabled evolution of M2WJ332 resistance, yet this did not occur for L1.1 with continued suppression of viral titre following the normalisation/bottleneck stage of the experiment. Interestingly, we have now tested archived virus from another intermediate passage (9), following the normalisation step. In the absence of inhibitors, distinct increases were apparent in the induced cytopathic effect for DMSO-, rimantadine- and M2WJ332-selected virus, whereas L1.1 selected virus cpe remains similar to the parental E195 strain. DL7-selected virus was unfortunately lost to follow-up so we have not included this data in the revised manuscript.

Figure 6c: I don't understand the second sentence of the 6c legend, particularly the second half: "Quantification of SP7 output titres generated from infection by the subsequent passage with quantified SP6 innoculae." Is it supposed to mean: "Quantification of SP7 output titres after infection by equivalent doses of SP6 innoculae?"

Yes, this is the correct interpretation – apologies that this was not clear, we have amended the text to that suggested by the reviewer.

Results describing Figure 6c: What is meant by the evolutionary bottleneck applied during passaging?

This refers to the normalisation of innoculae at passage 7 as discussed above, whereas previous iterations had involved bulk passage of 10% of the preceding culture volume.

Results describing deep sequencing: What was the range of conditions examined? Complete genome? If so, were there any other changes? All 5 compounds used for passaging? It’s odd that there were no changes for cultures with DL7, which showed resistance formation in figure 6D. What is the zanamivir mutation in NA?

Please see response above

Results describing synergy: It would be appropriate to acknowledge the “multiple comparisons” problem. Considering all 5 compounds tested, there are 24 possible synergy combinations you could have tested for. What is the likelihood that one would see two appearing to show synergy at the level observed and two showing antagonism out of that many tests even if there is no synergy or antagonism, i.e. randomly? It may be very high.

The reviewer is right to point out that our synergy observations may be affected by the “multiple comparisons” issue. Whilst this is a possibility, both software sets apply Bonferroni’s correction during the calculations of synergy scores. We also note that experiments in figure 7a display complex outputs where certain combinations of inhibitors induce antagonism, others synergy. This is unlikely to occur by random chance and may be linked to the presence of multiple peripheral sites and/or associated structural changes within the channel complex.

Discussion starting at the bottom of page 20 with “Eng195 chronic culture…”: This paragraph works hard to rationalize the peripheral binding site for WJ332, but given the beliefs of the field, the 2LY0 structure, and the resistance to S31N/V27A, it might be good to acknowledge that the failure to block the N31 TM is not necessarily pathognomonic for binding location.

 We have modified the discussion to include additional qualifying text.

PLOS authors have the option to publish the peer review history of their article ([what does this mean?](https://journals.plos.org/plospathogens/s/editorial-and-peer-review-process#loc-peer-review-history)). If published, this will include your full peer review and any attached files.


If you choose “no”, your identity will remain anonymous but your review may still be made public.


**Do you want your identity to be public for this peer review?** For information about this choice, including consent withdrawal, please see our [Privacy Policy](https://www.plos.org/privacy-policy).

Reviewer #1: No

Reviewer #2: No

Reviewer #3: Yes: Prof. David D. Busath, M.D.

*In compliance with data protection regulations, you may request that we remove your personal registration details at any time.* [*(Remove my information/details)*](https://www.editorialmanager.com/ppathogens/login.asp?a=r)*. Please contact the publication office if you have any questions.*
