# Supplementary material for: Site-directed M2 proton channel inhibitors enable synergistic combination therapy for rimantadine-resistant pandemic influenza
Source: PLoS Pathog. 2020 Aug 11;16(8):e1008716. doi: 10.1371/journal.ppat.1008716 (PMC7418971; doi:10.1371/journal.ppat.1008716)
Supplement: Attachment — Submitted filename: SG-SecondresponsetoreviewsPLoSPath2020v2.docx [file ppat.1008716.s015.docx]

**Dr Stephen Griffin**

**Associate Professor**

**Academic Lead for Laboratories**

School of Medicine

University of Leeds

St James’ University Hospital

Leeds, LS9 7TF

Tel: (+44)113 3438637

E-mail: [s.d.c.griffin@leeds.ac.uk](mailto:s.d.c.griffin@leeds.ac.uk)	10^th^ June, 2020

Dear Profs Lowen, Pekosz, Haldar, McFadden and Malim

Thank you for providing the second set of responses to our manuscript *“Site-directed proton channel inhibitors as synergistic combination therapy for rimantadine-resistant pandemic influenza”.*

We are naturally disappointed that reviewer 1 did not consider our rebuttal sufficient; we feel that the first review addressed the vast majority of the points raised in the second review. However, we are happy to amend the text further in light of the changes suggested; in particular, we accept that the wording concerning the lack of effect for DL7 in cell culture should be stronger. We note that many of the issues voiced by this reviewer concern the M2WJ332 compound, rather than the validity of the two binding sites per se.

We appreciate the interesting points raised by Professor Busath and have removed Figure S3E. We have retained Figure S3C for the time being and provided our rationale, yet we are happy to remove this if Professor Busath remains unconvinced.

We trust sincerely that incorporating these additional adjustments will render this manuscript acceptable for publication in *PLoS Pathogens*. Please do not hesitate to contact me if I can provide any further clarification.

Yours Sincerely

Dr Stephen Griffin, MA(Hons) Cantab, PhD Cantab

Thank you for your revisions. The manuscript has been reviewed by two of the prior reviewers. While their opinions differ, these reviewers each raised valid concerns regarding the revised text. We ask that you modify the text to address the remaining concerns of Reviewer 1, including how the present data relate to prior characterization of the binding site of M2WJ332 by NMR; role of position 27 in binding and resistance; interpretation of the electrophysiology data in Fig S3; implications of failure to select resistance to DL7; and rationale for the synergy noted when M2WJ332 is combined with either L1.1 or DP9.

As suggested by Reviewer 3, we also ask that you add commentary regarding the relationship of the present findings to those of Chizhmakov et al. 1996, and encourage you to consider removing Figures S3C and S3E.

Reviewer Comments (if any, and for reference):

Reviewer's Responses to Questions

Reviewer 1 (from attachment)

This revised manuscript by Griffin et al provides additional explanation that the newly discovered M2 channel blockers DP9 and DL7 target the allosteric site at the C-terminal domain, while compound L1.1 targets the N-terminal lumen. During the revision, the authors also provided additional experimental results showing that compound M2WJ332 inhibits proton conductance through the M2-N31 (CD) construct in lipid bilayer. It was suggested that a previously well-characterized compound M2WJ332 targets the C-terminal allosteric site, which contradicts conclusions drawn from previous solution and solid-state NMR results.

It was noted that majority of the issues raised from the previous round of review were not properly addressed. The authors provided no additional experiments such as solution NMR as suggested to support the proposed mechanism of action. The authors also failed to test the three compounds against additional influenza A strains to confirm the mechanism of action and antiviral activity. Therefore, the revised manuscript is still premature for publication.

We accept that we did not address this reviewer’s comments using the approaches suggested.

We attempted to make our reasoning for not doing so clear in the first response to reviewer’s comments. Specifically, the structural bias conferred by use of either TM (lacking the C terminal peripheral site) or CD (narrower lumen) peptides, further confounded by the lipid-mimetic environment, makes a valid structural comparison problematic in the absence of a lipid-borne structure for the full length protein. Solving multiple structures for combinations of peptides and inhibitors might go some way to address this, but this is beyond the scope of the present study; indeed, this issue was one of the major drivers that led us to undertake functional validation of compound binding to peptides reconstituted in lipids.

Unfortunately, the UK COVID19 lockdown prevented the testing of new inhibitors against multiple influenza strains. However, we did demonstrate that the L1.1 compound displays antiviral effect versus an engineered pH1N1 virus with a N31S mutation, reversing the resistance phenotype, in our first rebuttal. We are happy to include this data (with, or without the other prototype compounds) if the reviewer feels this is valuable. In this regard, several recent studies of M2WJ332-related M2-N31 inhibitors also focus upon a single virus background into which M2 mutations were introduced, and this was not always a clinically relevant strain, e.g. Musharrafieh *et al*., 2018; Musharrafieh *et al*., 2019.

Basically, there is no solid experimental evidence to suggest that the newly identified compounds DL7, DP9, and L1.1 are indeed M2 channel blockers, and the proposed mechanism of action that DP9 and DL7 target the C-terminal allosteric site was also not experimentally supported.

We naturally disagree. In addition to dye release and virological assays, we now include data showing that L1.1 also acts versus TM peptides in the electrophysiology assay, omitted from the first resubmission due to the lack of a comparison with CD peptides; this was again due to the onset of COVID19 lockdown. With the exception of DL7 (see below), we are confident that novel compounds act upon M2 *in vitro* and in cells. We emphasise that many more M2-active compounds were identified through our study and that subsequent focusing upon this small collection merely reflects a first-pass short-listing of compounds with different site preferences. As stated in the manuscript, these inhibitory compounds are only the first step towards development of new potential classes of antiviral.

Although the scientific community welcomes controversy, the conclusion needs to be supported by unambiguous experimental data but this study lacks solid experimental results. Here are the main points the authors need to consider:

We emphasise that our aim was never to court controversy, rather to reconcile discordant views upon the relevance of two potential binding sites upon the M2 channel complex that had previously only been interrogated using adamantane derivatives. Our intent was the expansion of avenues along which to reinvigorate rimantadine-resistant M2 as a drug target and to conduct comparative functional studies towards that aim.

1) The authors proposed that compound M2WJ332 binds to the C-terminal allosteric site based on the liposome flux assay results. This is the only piece of experimental result the authors provided to support this claim.

We refer the reviewer to the new data included in our first resubmission where we clearly demonstrate that M2WJ332 preferentially blocks CD peptides compared with TM in an orthologous electrophysiology set up (see fig3E, F and S3).

If the authors have done their literature review thoroughly, they should aware that the binding site of M2WJ332 is well characterized by both solution NMR and solid-state NMR. The N-terminal lumen binding mechanism was also supported by resistance selection. Do the authors believe that liposome flux assay is more reliable than the NOEs from NMR?

We do not doubt the NMR data referred to above, but, at the risk of further repetition, we point out again that these structures were derived using TM peptides and so incur structural bias.

As aforementioned, we do not disbelieve NMR data per se, yet we question the use of TM peptides for structural studies if you intend to discriminate between the two binding sites – the peripheral site is essentially absent. Thus, studies in full-length protein, ideally embedded within lipid, are needed. See below regarding expanded discussion of this subject.

Second, if M2WJ332 indeed binds to the C-terminal allosteric site as the authors claimed, how could the authors explain that the N-terminal lumen V27A mutant causes drug resistance? The V27A mutant was selected from earlier studies as well as the authors’ own study, but the authors seem to skip the explanation.

We have expanded our discussion of V27A resistance mechanisms and channel structures, as advised by the editors.

We included extensive discussion around the discrepancies between V27A and peripheral drug binding in the previous version of the manuscript, stating clearly that V27 interacts with M2WJ332 both in NMR structures, as well as in docking predictions within the lumen. However, we also noted the study from Pielak *et al*., which demonstrated that V27A and the S31N mutation destabilise M2 channel complexes, providing an additional mechanism whereby these changes might mediate resistance.

Apologies, we have been unable to find another study investigating M2WJ332 resistance. A related compound induced a V27I change in the channel complex (Musharrafieh *et al*., 2018). Other related compounds also derived alternative resistance mutations (Ma *et al.*, 2016). We would be grateful if the reviewer might clarify this point and apologise if we have missed a key reference.

Interestingly, a recent study has shown that resistance to another M2WJ332 analogue can arise via an L46P mutation, located within the peripheral binding site (Musharrafieh *et al*., 2019). This mutation causes widening of the N terminal region of the M2 channel complex in the vicinity of V/A27 and S/N31. We interpret this as supporting the findings from the Pielak study, whereby these two regions of the channel complex are linked in terms of the overall structure of the channel, i.e. mutations in one region can affect the other. We have naturally included this study in our expanded discussion.

2) The newly provided electrophysiology data is Fig. S3 did not support the proposed mechanism of action. It only showed that M2WJ332 inhibited current conductance through the M2-N31 (CD) construct. No conclusion can be drawn from this result whether M2WJ332 binds to the N-terminal lumen site or the C-terminal allosteric site. How come the authors did not test the three other compounds DL7, DP9, and L1.1 in this assay?

Revised figure 3E showed that M2WJ332 inhibited the CD peptide in the new electrophysiology system. Revised figure 3F showed that this compound did not block activity of the TM peptide. Thus, our results from dye release assays were reproducible using an orthologous system.

We have now included data for L1.1 versus TM peptides, omitted previously due to not comparing with CD peptides due to the COVID lockdown. Similarly, we did not have an opportunity to test DL7 or DP9.

3) The antiviral activity of the three compounds DL7, DP9, and L1.1 was only tested in one influenza A strain Eng195 which is not sufficient to prove the mechanism of action and the antiviral activity. The authors should at least test these three compounds against one amantadine sensitive influenza A strain (M2 -S31).

As discussed above, L1.1 was tested against wild type E195, or with M2-S31, alongside compounds B and K, in our previous rebuttal and it showed antiviral activity in both cases. Time did not permit DL7 or DP9 to be tested. Again, we are happy to include this data if it is of benefit.

If the proposed mechanism of action is correct, luminal-binding compound L1.1 should not be active, while C-terminal allosteric inhibitors DP9 and DL7 should be active.

We do not understand this assertion. **At no point have we made a link between the presence of S or N at position 31 and the binding of newly identified compounds to the lumen or the periphery**. As mentioned above, L1.1 is active against viruses harbouring either M2-N31 or M2-S31.

Moreover, we tested several compounds derived from the screen versus the M2-S31 peptide and showed them to have equivalent effects. This data was not included in the manuscript as it employed older stocks of M2WJ332 and DP9 that had lost much of their activity; however, the preferential effect of M2WJ332 upon M2-N31 remains noticeable.

*Comparison of effects of the activity two peptides, 40 nM N31 CD and S31 TM. Compounds in 1 % v/v DMSO were preincubated with peptide in 5 % v/v MeOH, liposome subtracted end point values were normalised to peptide + DMSO. Data is the average of two independent duplicate experiments errors bars represent standard error. Paired t-tests were carried out, on raw values, to assess statistical significance between M2 + DMSO and each compound (* = p ≤ 0.05).*

4) The fact that the authors failed to select resistant mutants for DL7 is a strong evidence that DL7 is not a M2 channel blocker. This evidence argues against the authors’ own statement.

We agree that DL7 does not appear to exert a specific M2-targeted effect in cell culture. We have further clarified the discussion of this in the manuscript using clearer language to this effect. We note that several of the compounds listed in table 1 did not show antiviral efficacy in cells despite activity *in vitro*, which could be due to several factors. We speculate that DL7 might cause a transient insult to MDCK cells, leading to dose-dependent effects upon infectivity during EC50 assays, whereas cells in long-term culture are able to adapt to this compound.

5) “Remarkably, combinations of M2WJ332 with either L1.1 or DP9 yielded synergistic reductions of viral titre (Figure 7a).”

Comment: if M2WJ332 and DP9 bind to the same luminal site, how could they achieve synergistic effect? Any logic?

Our data support that M2WJ332 and DP9 bind peripherally, rather than to the lumen. As described in the previous rebuttal:

*“We attribute this to the presence of up to four potential binding sites upon the M2 channel periphery. Accordingly, mild antagonism occurred when higher concentrations of M2WJ332 are present, suggesting a switch from a co-operative to a competitive process as these sites become increasingly saturated. We have added text to the discussion to explain this hypothesis.”*

We have added additional text to this aspect of the discussion for clarification.

6) Figure S5. Metabolic activity of M2-N31 inhibitor treated cells.

Comment: this should not be called metabolic activity, it is cellular cytotoxicity

We disagree. Tetrazolium dyes are processed by NAD(P)H dependent reductases into the insoluble formazan. This may be affected by processes extraneous to cellular cytotoxicity, and so is only to be taken as a proxy measure of cell viability. Notably, MTT assays can often give readings higher than controls, reflecting such perturbations.

**Part I - Summary**
Please use this section to discuss strengths/weaknesses of study, novelty/significance, general execution and scholarship.


Reviewer #1: Please check the comments in the attachment.

See above.

Reviewer #3: Griffin and colleagues have responded cogently to virtually all of the reviewers' comments, and have improved the paper appropriately. The biggest addition is that of bilayer experiments with the peptides.

We thank Prof Busath for this positive comment.

They are encouraging in one way and discouraging in two ways.

On one hand, they appear to illustrate drug blockability of peptide-induced conductance for M2WJ332 vs M2-N31, esp CD.

On the other hand, they must not have been successful for the novel drugs (not mentioned).

As aforementioned, we have now included data on L1.1 activity versus the TM peptide. It is frustrating that time did not permit experiments on the CD peptide, but this experiment does at least control for the TM peptide being blockable in this system, as noted for CD in above comments.

Furthermore, the supplemental figure illustrates a decades-old dilemma with M2 expression in planar bilayers: namely that although the channels were shown to be exquisitely selective and to give sub-fA currents for protons in mouse erythroleukemia cells (Chizhmakov et al 1996), and are readily blocked in transfected Xenopus oocytes (e.g. AMT blocks WT), they have not been found to be blocked (again, WT by AMT), had pA currents, and in the reviewer's lab (unpublished) to be non-selective among cations. This discrepancy has traditionally been explained (by the reviewer at least) as a possible result of modest (unmeasurable) surface tension in suspended bilayers deriving from the suspension from the torus, but the new results of single channel currents in the supported bilayers presented in Figure S3C rule out the explanation of modest surface tension strain induced channel over-opening because supported bilayers would be expected to have ~zero surface tension, much like vesicles and cells.

We thank Prof Busath for raising this point as it is often overlooked; we have now included discussion of this issue in the revised text.

We agree that the Chizhmakov paper is a rare example where proton conductance was investigated in mammalian cells. However, the assertion that single channel conductance lay within the fA range in this paper was an estimate extrapolated from current variance using large electrochemical gradients, rather than a (challenging) direct measurement. Bulk channel activity caused currents in the low pA range as we and others have seen for single channels in suspended bilayers (Duff & Ashley, 1992).

We agree with Professor Busath that M2 (as well as other viroporins) appears capable of conducting ionic species in addition to protons, although whether this is relevant to biological function appears doubtful for the most part. We had not considered the bilayer surface tension aspect of this, but the formation of our supported bilayers between the two gels results in a compressive pressure that presumably generates a degree of surface tension in the system.

If we assume that M2 peptides behave similarly in our dye release assays, it is interesting to note the discussion from the previous revision whereby reviewer 1 commented upon the relative concentration of inhibitors required to suppress channel activity. We interpreted this as being due to the vigorous activity that we see for M2 peptides within this system, requiring higher inhibitory concentrations to achieve blockade. However, higher inhibitory concentrations may also conceivably reflect the concerns expressed above.

Nevertheless, rimantadine and other compounds are able to block M2 in dye release systems, albeit at relatively high concentrations, and peptide activity is also responsive to external pH. We unfortunately did not test Rimantadine versus the S31 peptide in the bilayer system as this may have been informative in this regard, yet given that M2WJ332 and L1.1 work at sub-micromolar concentrations we predict that the system might resemble the surface tension in mammalian cell membranes.

Ultimately, affirmation of whether the systems used in this, or other studies, represent a relevant model of M2 activity that translates to being able to exert antiviral effects in culture remains to be confirmed. This will only be achieved upon demonstration of inhibitor target engagement by genetic (resistance) and/or biochemical/biophysical techniques in the context of virus-infected cells or animal models. For now, we can say that the combination of rational structure-based enrichment with screening via dye release and virological assays has generated biologically active compounds that form a promising platform for M2 antiviral development focused upon two separate targets.

The blockability illustrated in the bar plots of Figure 3E and 3F are very gratifying, and they claim to represent 3 experiments with small standard deviations. The level of conductance decline displayed in Figure S3E is gratifying. One wonders if over the course of the block process the single-channel level was attained and then complete block ensued. Figure S3E shows only a one-second trace, so the reader can't tell if the "blocked" trace was taken between single channel currents, or if all channels were blocked. In any event, the pA currents in Figure S3C recall haunting years of controversy in this reviewer's lab.

Higher peptide concentrations were used in drug inhibition studies for this exact reason – apologies if this was not clear, we have amended the methods and figure legend. Thus, the blockage trace in figure S3E does indeed show that all peptide channels within the bilayer are blocked.

I think the authors would be wise to remove Figures S3C and S3E - they arouse unfriendly reactions unnecessarily. Also, the x-axis in Figure S3B needs labeling and the and peaks should be labeled with associated membrane potentials, instead of just numbered. (Note that peak number 3 falls off the left of the plot into the axis label where there is no peak). If the authors want to state that they observed pA channels that they can't explain given the high selectivity and low maximum single channel conductance observed by Chizhmakov et al 1996, that would maintain scientific integrity AND make the case for channel structure dynamics that they want to suppose underlies dye permeation. I note, however, that such channel dynamics and corresponding fluctuations in selectivity would argue against the Chizhmakov findings, which should also be duly noted.

Apologies, we have re-labelled figure S3B.

We are willing to remove Figure S3E as suggested by the reviewer. However, we prefer to keep S3C if this acceptable. Whilst conflicting with the results of Chizhamakov, it gives a similar level of conductance for M2 peptide single-channel events observed by Duff and Ashley, 1992 (<https://doi.org/10.1016/0042-6822(92)91239-q> ) and relates to the discussion of this point above and in the revised manuscript.

Finally, it is troublesome that the authors suppose that the gel compartment equilibrates in peptide and drug concentration on the one-hour time scale. With typical diffusion coefficients in unstirred gel, Fick's laws would predict weeks, not hours, for the equilibration time of a 6 mM diameter well loaded from the side.

This is an excellent point. Prof Busath may be correct that it takes longer than 1 hr to reach a fully equilibrated state.

However, during assay optimisation we tested both 3% and 1% agarose, the former taking a long time (overnight or longer) to observe current. 1% agarose greatly reduced this time, leading to the experiments as stated. Moreover, whilst the well diameter is indeed 6 mm, the top electrode was placed very close to the gel/buffer well boundary, i.e. the interface bilayer is very close to the boundary. Thus, peptides were required to diffuse for <1 mm rather than 6 mm, which also influenced the time necessary for equilibration.

We have made appropriate changes to the methods section to clarify these points.

**Part II – Major Issues: Key Experiments Required for Acceptance**
Please use this section to detail the key new experiments or modifications of existing experiments that should be absolutely required to validate study conclusions.

Generally, there should be no more than 3 such required experiments or major modifications for a "Major Revision" recommendation. If more than 3 experiments are necessary to validate the study conclusions, then you are encouraged to recommend "Reject".


Reviewer #1: Please check the comments in the attachment.

Reviewer #3: (No Response)

**Part III – Minor Issues: Editorial and Data Presentation Modifications**
Please use this section for editorial suggestions as well as relatively minor modifications of existing data that would enhance clarity.


Reviewer #1: Please check the comments in the attachment.

Reviewer #3: Legend S2 needs number correction (it is called S3).
Significant figures for numbers need to be limited appropriately in the new material, and probabilities given as hypothesis test values (e.g. <0.05, as if the significance level threshold, alpha, had been preset).

Apologies, these issues are now rectified.

PLOS authors have the option to publish the peer review history of their article ([what does this mean?](https://journals.plos.org/plospathogens/s/editorial-and-peer-review-process#loc-peer-review-history)). If published, this will include your full peer review and any attached files.


If you choose “no”, your identity will remain anonymous but your review may still be made public.


**Do you want your identity to be public for this peer review?** For information about this choice, including consent withdrawal, please see our [Privacy Policy](https://www.plos.org/privacy-policy).

Reviewer #1: No

Reviewer #3: Yes: Prof. David D. Busath, M.D.

Figure Files:

While revising your submission, please upload your figure files to the Preflight Analysis and Conversion Engine (PACE) digital diagnostic tool, [https://pacev2.apexcovantage.com](https://pacev2.apexcovantage.com/). PACE helps ensure that figures meet PLOS requirements. To use PACE, you must first register as a user. Then, login and navigate to the UPLOAD tab, where you will find detailed instructions on how to use the tool. If you encounter any issues or have any questions when using PACE, please email us at [figures@plos.org](mailto:figures@plos.org).

Data Requirements:

Please note that, as a condition of publication, PLOS' data policy requires that you make available all data used to draw the conclusions outlined in your manuscript. Data must be deposited in an appropriate repository, included within the body of the manuscript, or uploaded as supporting information. This includes all numerical values that were used to generate graphs, histograms etc.. For an example see here: <http://www.plosbiology.org/article/info%3Adoi%2F10.1371%2Fjournal.pbio.1001908#s5>.

Reproducibility:

To enhance the reproducibility of your results, PLOS recommends that you deposit laboratory protocols in protocols.io, where a protocol can be assigned its own identifier (DOI) such that it can be cited independently in the future. For instructions see [http://journals.plos.org/plospathogens/s/submission-guidelines#loc-materials-and-methods](http://journals.plos.org/plospathogens/s/submission-guidelines)

*In compliance with data protection regulations, you may request that we remove your personal registration details at any time.* [*(Remove my information/details)*](https://www.editorialmanager.com/ppathogens/login.asp?a=r)*. Please contact the publication office if you have any questions.*
